# An Abolitionist Vicious Circle: Slaving, Antislavery, and Violence on the Shores of Lake Tanganyika at the Onset of Colonial Occupation

**DOI:** 10.1080/0144039X.2024.2349369

**Published:** 2024-05-07

**Authors:** Benedetta Rossi

**Keywords:** East Africa, Tanganyika, Congo Free State, slavery, abolitionism, violence, Catholic antislavery

## Abstract

In the late nineteenth century, abolitionists felt entitled to use all possible means to save the African victims of the slave trade. As European imperialism rose, abolitionism legitimized interventionism. This article explores how a major humanitarian movement could sanction colonial occupation and the violence that accompanied it. It also examines the position of African slaveholders who resisted the entrenchment of European rule and defended an order in which slavery was common. It focuses on two main actors: French Captain Leopold Joubert, Catholic royalist and former pontifical Zouave who supported Cardinal Lavigerie's Missionaries of Africa and Belgian King Leopold II's allegedly abolitionist endeavours; and Tippu Tip, a trader and slaver who, like Joubert, worked for self-styled abolitionists such as Leopold II and the Sultan of Zanzibar. The connected microhistories of these men show how the international problematization of African slavery fuelled both European imperialism and anti-colonial resistance, while also creating circumstances in which enslaved persons emancipated themselves. The article investigates the moral perceptions of individuals whose sense of self was predicated upon values embodied by Europe's monarchies, the papacy of Rome, and the sultanate of Zanzibar. Faced with what they perceived as existential threats to these institutions, they responded with rising radicalism.

## Introduction

This is the story of a vicious circle. In the second half of the nineteenth century success in fighting the slave trade was one of the main currencies in which moral capital was measured at the global level.^1^ Popes, kings and sultans championed the abolitionist cause. For all their claims to act as liberators, they were driven less by concern for the freedom of Africans than by apprehension about the uncertain future of their own power. Contingency matters: in moments of crisis institutions and individuals respond with escalating dogmatism to the challenges that confront them. Institutions threatened by new ideas and new values demand intransigence, loyalty and self-sacrifice as they struggle for self-preservation; and individuals in crisis suspend ratiocination and rely on institutions to make decisions as they seek to salvage the old ideas and values that give meaning to their life.^2^

The institutions in question are the papacy of Rome, the monarchies of France and Belgium, and the sultanate of Zanzibar; the people are popes, kings, sultans, soldiers, and believers. The Vatican’s power had shrunk, European kings and emperors were losing credibility in the name of liberal ideals, and Muslim Sultans were threatened by Europe’s rising imperialism. In these circumstances, faltering institutions demanded unquestioning loyalty, and their members committed acts that would have been repugnant to them had they not been carried out in the name of God, King, and Country, driven by a sense of urgency. What motivated abolitionists to harm and coerce others in their struggle against slavery in foreign lands? How did they justify their actions to themselves and to others? And, vice versa, what motivated those who owned and trafficked slaves to defend a system that abolitionists deemed barbaric? What gave legitimacy to slave-owning, slave trading, and anti-abolitionist positions?

This article explores these questions by focusing on two men, their networks, and their worlds. The lives of Leopold Joubert (b. 1842, d. 1927) and Hamed bin Muhammed el Murjebi, also known as ‘Tippu Tip’ (b. ca. 1840, d. 1905) became intertwined in the lands surrounding Lake Tanganyika. For a while, they acted as *de facto* kings of large territories and eventually the Belgian King Leopold II gave them official roles in the administration of the Congo Free State. Joubert worked in support of the most influential Catholic abolitionist of the times, Cardinal Charles Lavigerie, who responded directly to the Pope in Rome and dreamed of establishing a Christian kingdom in the heart of Africa. In the 1880s, Lavigerie saw Joubert as the man who could rule this kingdom. Joubert, devout Catholic royalist and military man, established a confederation of local African chiefs who accepted his authority and agreed to collaborate with Christian missionaries in exchange for support and protection. Many converted to Christianity and vowed to stop engaging in the slave trade and fight ‘Arab slavers’. The latter included warlords supported by rulers who appeared to champion abolitionism. Both Leopold II and Zanzibar’s Sultan Sayyid Barghash placed large regions surrounding Tanganyika under the command of Tippu Tip, a powerful trader, caravan leader, and slaver.

Like moonlight, the power of Joubert and Tippu Tip was reflected from other sources. They weren’t absolute rulers, but acted on behalf of some of the highest politico-religious authorities of their times. They risked their lives, endured enormous hardship, and unleashed violence in the name of ideals they firmly believed in. These ideals were fundamentally religious: both Joubert and Tippu Tip served superiors whose actions they considered guided by God. Take these rationales away, stop them, as it were, from irradiating their exalted principles on these men’s words and deeds, and Joubert and Tippu Tip are but two delinquents using violence to subdue enemies and force locals into conformity with their respective agendas. The institutions they served, and the principles that these institutions incarnated, lent legitimacy to their actions. In all this, their respective predicaments both separated and united them.

Religious affiliation divided them. Joubert saw Tippu Tip as a Muslim slaver and, as such, a representative of the group whose power he was meant to crush. Tippu Tip’s attitude toward European abolitionists in his land was more nuanced. Aware of their military might and of his Sultan’s complex relation with them, he acted diplomatically. Joubert and Tippu Tip’s involvement in the antithetical projects of their superiors set them apart in events that irreversibly altered East Africa’s regional politics. And yet, they resembled each other in their efforts to defend institutions that were about to succumb to the forces of modernity. Their awareness of the dangers threatening the causes they served intensified their commitment. Their worlds were still threatened by the usual enemies: familiar unbelievers, barbaric pagans, and co-religionists whose interests clashed with their own. But they were also menaced by secular-liberal ideas which weren’t new per se, but were increasingly hegemonic.

The just-war logic that had underpinned fights in the name of Christianity and Islam for centuries now competed with new secular rationales that applied a symmetric moral logic to all warring parties.^3^ Henry Dunant, Swiss banker and colonial entrepreneur born to a wealthy family with business in French Algeria, wrote *A Memory of Solferino* based on his visit of the field after the battle.^4^ Dunant’s book influenced the first Geneva Convention of 1864, a pillar of modern international humanitarian law, and a founding text of the International Red Cross.^5^ His ideas did not differentiate between winners and losers on moral grounds. This new logic was pitched against the asymmetric just-war rationale that had dominated in the Christian and Islamic worlds since Medieval times and still informed the reasoning of many Christian and Muslim believers.^6^ In the same year of the congress at which the first Geneva Convention was developed, Pope Pius IX (Giovanni Maria Mastai Ferretti) published a ‘Syllabus of Errors’ as an appendix to the encyclical *Quanta Cura*, which anathemised ideological developments of the nineteenth century that he saw as opposed to the ‘one true way’ espoused by Catholic conservatives.^7^ Dunant’s views were one important facet of the new order of things to which Joubert’s and Tippu Tip’s ideals would succumb, for this is how this story ends. But not without first causing enormous bloodshed in its unfolding.

This article enquires into the moral justifications for slavery-related violence: which forms of violence were perceived as civilizing, which ones as barbaric, why, by whom, and with what consequences? It explores decisions to coerce less for their consequences for enslaved Africans than for what they reveal about what counted as acceptable, even necessary, violence in the eyes of men *whose worlds were under threat*. The celebration of some acts of violence as heroic valour and self-sacrifice, and the condemnation of others as savagery and rebellion depended on which rationales were mobilized when violence was imagined, witnessed, unleashed, or assessed. A first distinction is between abolitionist and anti-abolitionist (or pro-slavery) positions. But subtler distinctions are just as important.^8^ There were many ways of being an abolitionist and many ways of opposing abolitionism. People’s attitudes toward (anti-)slavery reveal what was at stake for them existentially when new approaches to sovereignty and hierarchy replaced old ones.

## The Worlds of Leopold Joubert and Tippu Tip

Joubert and Tippu Tip were men of the *ancien régime.* They belonged to separate *anciens régimes*, the French Catholic monarchy and Zanzibar’s Islamic Sultanate, which shared some commonalities. For both, political legitimacy followed religious criteria; wealth was distributed according to God-given hierarchies; and individual freedoms made little sense, as people were expected to accept the innate roles that a divine justice attributed to them. These criteria were challenged by new moral principles. Increasingly, political authority acquired legitimacy through consent; wealth ought to derive from freely contracted labour; and liberty was starting to be seen as a right of individuals and peoples. Eventually not men serving God and King, but colonial administrations manned by bureaucrats would replace the likes of them in Africa. The old principles that had supported the unity of religious and temporal power, and subjectivities defined more by interdependence than by individual autonomy, were losing ground to new values.

The same wave of changes engulfed both Joubert’s and Tippu Tip’s worlds with different consequences. The ideological revolution that eventually led to the spread of abolitionism worldwide implied a redefinition of slavery from a legitimate institution to a moral aberration.^9^ Abolitionism transformed the meaning of ‘slave’ from a diversified range of statuses and conditions that could justly be imposed on the legally enslavable to an illegal condition of social death conceived as internally homogenous and practically permanent, an intolerable injustice that turned all slaves into dehumanized disposable chattel. At least since the late seventeenth century the horror and magnitude of the trans-Atlantic slave trade had fuelled critiques that progressively yielded both a semantic narrowing of ‘slavery’ and the global spread of an abolitionist ideology that presupposed slavery’s new, narrower, meaning.

In the second half of the nineteenth century the work of prominent British abolitionist David Livingstone intensified efforts to suppress slavery and the slave trade in eastern Africa.^10^ European rulers who had joined the abolitionist crusade late, such as the Pope in Rome and the Catholic King of Belgium, endeavoured to prove their abolitionist credentials, as this was the prime currency of prestige in the foreign policy of Western powers. Lavigerie’s newly founded religious order, the Missionaries of Africa (also known as ‘White Fathers’), embarked on an antislavery crusade at a time when the Pope had suffered a fatal defeat at the hands of supporters of Italy’s unification and independence. Only few decades prior to this, the attitude of the Vatican toward slavery had not differed substantially from that of the moral authorities of Islam: slaveowners were urged to be generous toward their slaves, treat them humanely, and manumit them if they could. Cruelty toward slaves was condemned, not slavery itself, an age-old institution that had been tolerated by pious men on condition that it be humanely practised.

Pope Leo XIII’s (Gioacchino Pecci) encyclical *Catholicae Ecclesiae* on slavery in the missions was released on 20 November 1890. It stated that the Church ‘from the beginning sought to eliminate slavery’.^11^ But Vatican studies developed in preparation of the encyclical argued that liberation would have been detrimental to the enslaved in earlier times.^12^ Like the arguments of many contemporary Muslim authors, Catholic anti-slavery had tolerated the paternalist approaches to slavery that had contributed to the legitimation of Christian and Islamic slave ownership in the past.^13^ But in the second half of the nineteenth century, the Vatican aligned itself with the *doxa* of the times and changed the tone, if not the substance, of its older discourse.^14^ Cardinal Lavigerie played a fundamental role in bringing the Catholic Church’s approach to slavery up to date with that of the global abolitionist movements that had an Anglophone and Protestant genealogy.

Anti-slavery could restore the spiritual leadership of the Church after its temporal power had virtually ended and its political legitimacy had been fundamentally questioned not only by Muslims or Protestants but also by liberal insurgents, including Catholics among them.^15^ Catholic leaders thought that their actions in favour of African slaves attested to their moral superiority over the abolitionist approaches of ‘liberals’ and Protestants. Lavigerie portrayed these other abolitionists as driven by mundane interests.^16^ Furthermore, like many other Christian authors, he held Islam responsible for the lingering vitality of the African slave trade.^17^ The caravans of suffering slaves led by ‘Arab’ traders were a trope in European denunciations of slavery in Africa, a trope that Lavigerie contributed to promulgate in powerful orations delivered from the pulpits of Europe’s cathedrals. For example, his lecture on African Slavery in St. Sulpice in Paris described how ‘Arab’ and mixed ‘Arab-African’ slavers attacked African villages and terrorized defenceless populations. It cited evidence provided in the reports of European explorers, officers, and missionaries based in Africa. Lavigerie’s detailed descriptions of the cruel treatment of slaves in Muslim societies were reproduced in all the main French and European newspapers.^18^

Muslim intellectuals were offended by these accusations and some of them decried the hypocrisy of Christian arguments. For example, the General Governor of Ankara Abedin Pasha (of Greek Albanian origins and educated at Athens University) wrote an open letter in the Ottoman newspaper Vakyt which challenged Lavigerie’s position.^19^ Ahmed Chafik Bey, Egyptian scholar, former political attaché of the Khedive, and Secretary Particular of the Egyptian Minister of Foreign Affairs, published a study entitled ‘Slavery from the Muslim Point of View’. The preface’s first sentence demonstrates that the author saw his study, dedicated to the Khedive, as a rebuttal of Lavigerie’s arguments:

On 1 July 1888 I had the chance to attend a conference by Cardinal Lavigerie in the Church of St Sulpice in Paris. The main objective of this conference was to denounce the horrors of the slave trade in Central Africa and of slavery in Muslim countries to the Parisian public. His Eminence, not satisfied to blame Muslims for this, imputed such horrors to the religion of the Prophet … ^20^

In the previous decades, Egypt’s khedives and other North African and Middle Eastern Islamic rulers had taken steps to abolish the slave trade and suppress slavery. These efforts were now denounced as insincere and insufficient.^21^ Whatever the pertinence of Europe’s critiques, they weren’t disinterested. In the late 1880s Muslim rulers realized that previous efforts were not going to suffice to protect them from Europe’s growing imperialism. Failure to be seen as supporting abolitionism could cost them everything. This realization accelerated their endorsement of the abolitionist cause, but eventually did not suffice to protect African rulers from Europe’s invasion.

Wealthy Muslims who had owned and traded slaves and who thought they followed Islamic precepts on slavery did not cease to be at peace with their conscience overnight. Slaveholders would not suddenly think of themselves as monsters because the likes of Livingstone and Lavigerie – infidels and potential usurpers in their eyes – portrayed them as such. Throughout the whole nineteenth century slavery had been a common institution that permeated all aspects of life, with multiple categories of slaves occupying a broad range of positions in society. East Africans discussed moral and immoral attitudes toward slaves but didn’t question slavery’s legitimacy as an institution.^22^ Abolitionist ideas had a different appeal to different groups of Europeans and Africans disposed to perceive slavery, slaving, and slaves in different ways.

The expansion of trade in commodities sold on global markets had led to an intensification of slaving from the Swahili coast to Lake Tanganyika in the nineteenth century.^23^ Intensified slaving fed the militarization and ‘privatisation of violence’ that characterised the rise of politico-commercial-military systems headed by men like Tippu Tip and Mirambo.^24^ Enslaved persons worked at the bottom ranks of local commercial agriculture and trade institutions, including the large caravans that brought products from the interior to the coast.^25^ Such caravans also contained human commodities that met a demand for slaves in coastal regions, North Africa, the Middle East and across the Indian Ocean. Enslaved people were transferred through tributary arrangements between polities.^26^ Debt and penal slavery are well-attested in the Swahili coast’s interior, where killers or adulterers could be sold into slavery in punishment for their crimes.^27^ Defaulting debtors could be pawned, or forced to provide pawns, who in turn were vulnerable to enslavement.^28^ Some of these practices went against Islamic laws regulating slavery, but were customary practice.^29^

Islamic jurisprudence had been regulating trade and slavery in this region since medieval times.^30^ Muslims around Lake Tanganyika followed shared legal and commercial norms. Muslim legal specialists provided guidance to slave-owners on what counted as correct relations with different categories of slaves. Bankers extended credit to long-distance traders (and in the nineteenth century also to European travellers and explorers).^31^ Tippu-Tip’s caravan expeditions are a case point: underwritten by ‘*waraqa*’ debts contracted with the Gujurati banker Khoja Tharia Topan, they made available to Tippu-Tip advances in commodities (specific types of cloth and other items functioning as currencies) that he needed to pay porters and buy ivory, slaves, equipment (e.g. food, guns) and gifts for partners and gatekeepers along the trade routes.^32^ Slaves were not usually pledged in Swahili trade, but other goods were pledged to finance the operation of caravans that transported ivory, slaves, and other valuables.^33^ Occasionally, slaves were pledged, too.^34^

Unless specifically aimed at the recruitment of male workers, wars tended to yield male casualties and female captives. The sexual and reproductive functions of girls and women made them valuable not just as workers but primarily as concubines and mothers. Women were also distributed as gifts that cemented relations between male political allies and commercial partners.^35^ Tippu Tip’s autobiographic text, known as the Maisha, contains frequent references to enslaved women.^36^ Although the numbers mentioned in it may be exaggerated, the text reveals the author’s attitudes toward female slaves. For example, once a group of Tippu Tip’s associates joined him after going on an expedition and brought back with them 600 enslaved women captured in nearby villages. The chiefs of the injured villages then established a connection with Tippu Tip’s father and sent a delegation, which included Tippu Tip’s father, to the camp of Tippu Tip and his party to negotiate peace and the women’s return. Tippu Tip agreed, but required compensation for his men who had been killed, which he received in the form of about 15 *frasilas* (ca 240 kg) of ivory.^37^ The Maisha is filled with similar occurrences: an affiliate of Tippu Tip reconnoitred enemy territory and came back with about 1000 captive men and women and 2000 goats.^38^ Elsewhere ‘all the enemy died and we set fire to their villages as well as taking 400 women prisoners’.^39^ Again, once Tippu Tip and his men fought against a group which only had spears and arrows as weapons; he argues that they left 700 or more men of the enemy dead and ‘took captive more than a thousand women and countless goats’.^40^ A chief who wished to establish an alliance with Tippu Tip asked: ‘I wish for friendship with Tippu Tip, what does he want, women or ivory?’^41^

In Tippu Tip’s writings enslaved women’s value is entirely relational: it is a function of their ability, or inability, to serve the interests of men. However, female captives who became concubines could acquire respectable roles in the families of their owners. For slave women, a sexual or conjugal union with their owner was the main avenue to security. The story of Tippu Tip’s grandmother is a case in point. Tippu Tip tells of how his grandfather had bought a beautiful captive woman from Utetera who had been sold in Urua: ‘He took her to the coast and made her his concubine, where she bore my mother’. He explains that Darimumba, his grandmother, used to tell him that in her country she was a member of the royal house; that there were much ivory and many people, and that her brother was ‘a powerful chief called Kasongo Rushie Mwana Mapunga, and that all the Watetera and Wakusu were their people’.^42^

Although kinship ties were brutally severed for captives upon enslavement, kinship connections from their life before enslavement were occasionally acknowledged by the free members of the communities in which slaves were forcibly integrated. Tippu Tip argues that because of his enslaved grandmother’s connection to an earlier chief, he himself was welcomed as a chief in Utetera: Kasongo voluntarily relinquished power to him and accepted him as a co-ruler. As a friendly gesture, Tippu Tip returned his people (whom he had captured) to him. This anecdote may or may not have actually happened as narrated by Tippu Tip, but it sheds light on local perceptions of the status of enslaved women and slaves more generally. Though slave-owners only seldom expressed concern for the sufferings they inflicted on the enslaved, especially those newly captured, they didn’t dehumanize them. Once dependent on particular slaveowners, enslaved persons acquired a specific rank in a hierarchy of slave statuses in which belonging *as slaves* was possible and loyalty could result in social mobility within slave status and manumission by paternalist ‘masters’.^43^

Extant narratives of liberated slaves suggest that many were able to achieve substantial economic and social mobility in the second half of the nineteenth century. Some, both men and women, became emancipated by attaching themselves to Christian missions.^44^ Others followed Islamic avenues of manumission and tried to turn to their advantage ties of dependence from former owners. For example, Msabbah and Songoro were manumitted by Talib bin Abdullah, a wealthy man of Omani descent, in the 1850s. They obtained property either though gifts at manumission or as beneficiaries of *waqf* lands left for the benefit of poor Muslims.^45^ They then sold or used as security for credit this property to start investing in the caravan trade. They continued using the clan-name of their former master and benefiting from patronage by freeborn members of his clan. They became important ivory traders and interacted with the main local rulers and European explorers. Songoro constructed the first dhow that sailed on Lake Victoria in the mid-1870s. In the second half of the nineteenth century, Gujarati bankers made large numbers of loans to liberated slaves who worked and traded on their own account.^46^ Exemplary cases of self-realization through loyalty leading to manumission influenced how East African Muslims perceived both slavery in their society and European abolitionism. But the generalization that slavery was benign in these societies is unwarranted.

Much has been written about the ubiquity of violence in the regions surrounding Lake Tanganyika in the late nineteenth century, especially the Lake’s south-western shore, today’s eastern Democratic Republic of Congo.^47^ One thing is clear: even though enslaved persons had concrete options for social mobility, violence and slavery were inseparable. In the writings of travellers in East Africa, slaves are everywhere. They are captured, exchanged, sold, liberated, saved, gifted, married and put to work in all domains, including soldiering. For the majority of those enslaved and families who lost relatives to slavers, enslavement was a dreadful ordeal. Violence was required to capture, abduct, and coerce slaves. It was required, too, to free them, protect them, and fight enslavers. It was perpetrated against, for, and by slaves.

Different assessments of the circumstances of enslaved Africans nourished antagonism between the worlds of Joubert and Tippu Tip. In their respective attitudes toward slaves, both abolitionists and slave traders followed shared codes of conduct founded on ethical norms that oriented their life and reproduced hegemonic worldviews. When these norms were disputed by outsiders who radically rejected the whole apparatus that generated them, defence did not result in debate. Defence was emotional and aimed at protecting identity seen as threatened by external aggression to institutions which actors identified with. Those who stood most to lose were not just irritated but outraged: they denounced opponents not only as dangerous threats to themselves but as despisable threats to God and society. They were prepared to defend the previous order with their life. And indeed, their life was at risk, life as they knew it.

Fighting the horrors of slavery in Africa gave Christian abolitionists a renewed *raison d’être* that enhanced their self-righteousness. In turn, their commitment to this new crusade challenged East Africa’s Islamic hierarchies. And so it was that institutions sustained groups that sustained individuals who sustained groups who sustained institutions. That this could turn into a vicious circle of escalating violence should not surprise us. At stake was less the destiny of the enslaved than the abolitionists’ and slave-owners’ lifetime commitment to institutions that made them who they thought they were.

## How Networks Mobilized

Leopold Joubert and Tippu Tip belonged to political, religious, military and commercial networks too deeply reliant on imperilled institutions not to mobilize to salvage them. Trust followed blood-ties, real and fictive. On his father’s side, the earliest known ancestor of Tippu Tip’s family was Tippu Tip’s paternal great-grandfather Rajab bin Muhammad bin Said al-Murjebi, born in the Adam region of Oman and arrived in East Africa at the end of the eighteenth century.^48^ Rajab al-Murjebi had a travel companion from the al-Nabhani family that had been involved in Imamate politics in Oman and whose father had already conducted trade in the East African coast. Tippu Tip’s kin and network of allies who claimed Arab descent had Omani roots like the Sultanate they had been serving both in Oman and in East Africa. Tippu Tip was the third generation of his family to trade between the Indian Ocean coast and the East African interior, particularly the Unyamwezi region. Tippu Tip’s relatives held influential positions in ‘Arab’ centres along the main axes of caravan trade. Fictive kin could stretch to include all of east Africa’s ‘Arab’ networks who recognized the Omani Sultan as suzerain and followed the same ethos, even though they did not always act in unison: ‘All my Arab kinsfolk were fearful and suggested that we stay there until the following day. But I said to them: “what? Stay in the bush? Don’t be such cowards!” We set off in strength … ’^49^ Moreover, marriages between Arab and Swahili merchants and local rulers meant that solidarity could conjoin ethnically defined groups.^50^ European interventionism triggered resistance both within and beyond ethnolinguistic networks.

Similarly, Joubert’s networks comprised his mother and siblings, with whom he always corresponded; Catholic royalists mainly based in Brittany whose families had known each other for decades; and Zouaves who were mostly French. Joubert, like Tippu Tip, repeatedly demonstrated readiness to risk his life when others were reticent to do the same. He never lost awareness of his place in the religious-monarchic hierarchies that he and his ancestors served generation after generation. He supported the Church, popes, and monarchs whose power he believed to derive from divine providence.

Like Tippu Tip’s world, Joubert’s world now appeared vulnerable. Those who defended it belonged to families which had played the same roles for centuries. Their lives were rhythmed by Catholic sacraments and rituals. Their priests included relatives who made their careers in the Church and who christened, married, buried them, and mediated between them and their God. Shared religious mores and practices encompassed class divides, even though inequalities were reflected in the rankings of military-religious hierarchies. Joubert and Tippu Tip belonged in separate hierarchies. They mobilized within networks of higher- and lower-ranking actors to defend principles inherent in their respective hierarchies. While they acted individually, their actions acquired momentum in connection to those of others, some of whom they saw as authorities who had to be obeyed, while others they saw as subordinates who had to be guided. Hierarchies had ‘agglutinant’ properties that intensified individual commitment. Their members acted out of a sense of obligation to shared principles, awareness of their place in the hierarchy, and the requirements of loyalty and duty (of service, of command).

Key actors did not just *happen* to find themselves in the same places, following orders to join conflicts they were not personally invested in. They belonged to connected families which, for generations, had been serving the same authorities and ideals. The Vatican and Catholic elites had been forced in a defensive position and called upon trusted individuals. Xavier de Mérode, whose father Felix de Mérode-Westerloo had overseen war affairs for Leopold I of Belgium, was Pope Pius IX’s main advisor.^51^ De Mérode had started his career as a military man and had received the Legion of Honour for his actions against the Kabyle in Algeria in the mid-1840s. In Algeria he had met Christophe Louis de la Moricière, who commanded the Zouave corps composed of French and native Algerian soldiers enrolled in the French colonial army.^52^ When the Italian wars of independence menaced the temporal power of the popes, de Mérode advised the Pope to put de la Moricière in charge of reorganizing the pontifical army. Publicly opposed to Napoleon III, de la Moricière had found his Catholic faith in exile in Belgium where he resettled after Algeria. He proposed that the Pope recruit volunteers from Catholics abroad to expand the size of his army. Pius IX agreed, and hundreds of volunteers responded to the call.^53^ Originally named French-Belgian Tirailleurs, the international volunteers were eventually conscripted in a new corps of ‘Zuavi Pontifici’ founded by de la Moricière in 1861. In Brittany General Athanase Charette de la Contrie mobilized local volunteers for the papal cause.^54^ Athanase was a descendant of François Athanase Charette de la Contrie, one of the foremost anti-revolutionary fighters in the revolt in Vendée.^55^ Hence, in the 1860s a network of Franco-Belgian ultra-Catholic royalists whose ancestors had been among the main opponents of the French Revolution supported the Popes and what Papal power stood for: God-ordained political and temporal authority.^56^ Their position in society depended on the hierarchies that the revolution had overthrown.

The great questions of Joubert’s times concerned the moral justification for political authority and hierarchy, the place of religion in politics, and the tension between republicanism and monarchy. In Joubert’s youth, France’s July Monarchy (1830–1848) was followed by France’s Second Republic (1848–1852), Second Empire (1852–1870), and Third Republic (after 1875). Leopold Joubert’s grandfather had fought in the Chouannerie, a royalist counter-revolutionary uprising in Western France. He told young Joubert stories of glorious battles for God and King.^57^ From the age of 12 to the age of 16 Joubert attended secondary school at the college St Joseph d’Ancenis, where Christophe Louis de la Moricière was a celebrated alumnus. When news reached him about the call for volunteers of General Athanase Charette, he wrote to his mother asking permission to enrol with Charette’s volunteers.^58^ He embarked in Marseille on *Le Vatican* on 27 July 1860. In Rome he joined the French-Belgian corps commanded by the General Louis Aimé de Becdelièvre.^59^ This corps would form the core of the Pontifical Zouaves.^60^ Its characteristic uniform, modeled on the one of the Zouaves of Algeria, was recommended by de la Moricière as well adapted to the heat of Rome.

It is scarcely possible to understand the subsequent endeavours of Joubert and his Catholic peers in Africa without considering their fervour and failure in Rome in the 1860s. The first important battle fought by the papal volunteers, who had been arriving in the course of the previous months, took place in Castelfidardo on 18 September 1860. The Zouaves were a small minority of the 10,000 soldiers who fought on the side of the Pope commanded by de la Moricière. Overpowered by the Piedmontese army, they were defeated. De la Moricière was never to command a papal army again. Joubert, hit by a shot to his leg in Loreto and made prisoner by the Piedmontese, was cured in Fesi and sent back to France.^61^ He could have stayed home, but chose to return to Rome. At the age of 25 he was made Captain and in September 1870 he commanded the 6th company of the 1st battalion defending Rome at Porta Salaria against the Piedmontese. Joubert and other French Catholic royalists saw French identity as founded on Catholicism and the defence of the Holy See:

At Rome the regiment of zouaves … accomplished a French task in protecting and defending the independence and sovereignty of the Holy See. It is not for nothing that the statue of Charlemagne stands guard at the gates of Saint Peter at the Vatican.^62^

Pius IX had just promulgated the doctrine of papal infallibility at the fourth session of the First Vatican Council in July when the temporal power of the popes ended on 20 September 1870. Italian armies took Rome and achieved Italy’s unification. Joubert returned to France, where he joined General Charette and other former Zouaves in the newly constituted corps of the *Volontaires de l’Ouest.* The *Volontaires* fought at the battle of Loigny, which carried enormous symbolic significance for French Catholic royalists.^63^ After the defeat of Loigny, Joubert and other Zouaves refused to join the national army of the Third Republic because of their profound monarchical convictions.^64^ As it became impossible to continue fighting for the cause they believed in, their military life in Europe came to a halt. But new horizons opened for Joubert in Africa.

Lavigerie was Archbishop of Algiers and Apostolic Delegate for the Sudan and Sahara. Two caravans of his recently founded order, the Missionaries of Africa, had been massacred by Tuareg groups in 1876 and 1881, respectively.^65^ Catholic missionary penetration in Islamic West Africa faced serious challenges. This did not deter Lavigerie who, like the French military royalists, saw the missionary penetration of Africa ‘in terms of the early medieval experience of Christianity in his native France’.^66^ Pope Leo XIII succeeded to Pius IX on 20 February 1978 and authorized Lavigerie to send missionaries to East Africa via Zanzibar. The first caravan of the White Fathers split into two in Tabora, one branch destined to the shore of the Tanganyika Lake, the other to Victoria-Nyassa in today’s Uganda. It became clear that the missionaries’ chances to succeed would be improved if they relied on the support of devoted military men with experience in fighting and organizing missions in perilous environments.^67^

After the end of the Franco-Prussian wars, the former Zouaves and *Volontaires de l’Ouest* had remained in contact with each other. Leopold Joubert in his home of Mésanger was a close neighbour of General Charette in Couffé. The General employed Joubert as personal secretary and preceptor of his son.^68^ Charette, an old acquaintance of Lavigerie, recommended Joubert as a committed Catholic with the required experience and proven devotion. A meeting was arranged between Joubert, the General, and the Cardinal at which it was decided that Joubert would join the third caravan of the White Fathers.^69^ Joubert arrived in Algeria, his first trip to Africa, at the end of January 1880 when he was approaching 40 years of age. The volunteers recruited by Joubert followed a period of training at *Maison Carrée*, the White Fathers’ headquarters in Algiers. Joubert reached Ujiji on the eastern shore of Lake Tanganyika on 7 February 1882. Tensions with local ‘Arab’ elites were not yet evident and Tippu Tip was seen as an ally.^70^

This was no idle reverie. Tippu Tip was ‘the man without whose permission it is impossible to penetrate Africa’.^71^ He had supported many European traders, explorers, and missionaries. The antislavery activities of David Livingstone had done much to turn Europe’s attention toward slavery in the eastern part of the African continent, but at the end of the 1860s Britain had lost contact with Livingstone who was travelling in East Africa. Stanley was sent to find him, which he did on 10 November 1871 in Ujiji, where Tippu Tip had been helping him. Tippu Tip’s men found Livingstone, who ‘had almost been killed’, and brought him to Tippu Tip’s camp for protection.^72^ Tippu Tip had also supported Verney Lovett Cameron, who in 1874 asked him to accompany him on his trip and help him defend himself from the people of Nyangwe.^73^ Tippu Tip rearranged his plans to satisfy Cameron’s request and gave Cameron men who would accompany him.^74^ Cameron acknowledges all this in his own memoirs which confirm Tippu Tip’s account.^75^ Tippu Tip also tells of how Hermann von Wissman, hardly an admirer of East African ‘Arabs’, greeted him when he arrived in Tabora: ‘ … Everywhere I passed I mentioned your name and what I wanted became available. Now I’ve come here, without provisions or anything else. Give me goods, and I’ll repay you when I get to the coast’. Tippu Tip replied: ‘Agreed, whatever you want!’^76^

Tippu Tip’s generosity was essential to his sense of self. He emphasized it not only in his interactions with Europeans but also with other regional warlords, such as Mirambo and Muhammed bin Khalfan (known as ‘Rumaliza’). For example, when Rumaliza asked him for ‘a body of troops’ to fight against enemies, ‘I gave him rather more than 20 Waungwana [free men], men of the Mrima coast and more than 140 guns; together with slaves and their weapons and powder’.^77^ A few years later, when Rumaliza informed him that ‘the people of Lake Tanganyika’ (the White Fathers and Joubert?) were trying to deprive him of the areas of Urundi, Uvinza and Masenze.

He asked whether I could send him some slaves and weapons. I sent off to him 500 guns and men, each man with his own gun, and told him that I was on my way, saying that if he was at Uvinza I would meet him at Ujiji.^78^

Tippu Tip’s generosity was surely also opportunistic. Alliance with other ‘Arab’ warlords were vital in a world where reciprocal obligations could be life-saving. Power was measured primarily in terms of a person’s ability to control people; indebting others to oneself gave access not only to their good will, but also to the services of their dependents. As for Tippu Tip’s beneficence toward Europeans, it resulted in payment for his services and guns, which were essential to his commercial success. But his stated motive was that supporting Europeans conformed with the Sultan’s will. Since he could not hope to defeat Europeans militarily, Sultan Barghash sought to turn them into allies. Tippu Tip played a central role in this. Contemporary European sources frequently presented the actions of these two men as connected.^79^

The Sultan was the ultimate authority in Tippu Tip’s world, and obedience to the Sultan was what made his activities and success possible in the first place. For example, when Stanley returned to Zanzibar to lead the Emin Pasha’s relief expedition, Tippu Tip helped him by mobilizing the men’s loyalty toward himself and the Sultan.^80^ Locals disliked Westerners and would have left Stanley alone. Stanley’s party went to see Tippu Tip overnight: ‘This European is mean; he counts everything out; he doesn’t even give us clothes … ’ Tippu Tip went to Stanley, got goods from him, and distributed them to the men, who then agreed to follow Stanley.^81^ Spiritan Father Alexandre LeRoy, a critic of Lavigerie and Leopold II’s abolitionist approaches, wrote that if Stanley had not had Tippu Tip as his ally, but as his foe, he would have surely died.^82^ Tippu Tip’s support to Stanley was forthcoming in spite of the local hostility against Europeans generally, and Stanley specifically:

people were more outspoken in trying to dissuade me, and derided me ‘What, going with a European, have you lost your senses? You’re mad, will you then become a European? Yet you’re not needy, why then? You have your stock of ivory, why then follow an Unbeliever?’ I told them: ‘Maybe I am mad, and you that are sensible, keep to your own affairs’.^83^

Anti-European sentiments were widespread. But Tippu Tip devised a subterfuge to help Stanley. When Stanley’s men declared that they wouldn’t continue travelling with Stanley if Tippu Tip left, Tippu Tip instructed Stanley to speak harshly to him in front of his men and say:

Look here, if you return, all my men will insist on returning. Well, my business is the business of the government, and we are in agreement with Sayyid Barghash. If my men return, then when I return I shall tell Sayyid Barghash that it was Hamed bin Muhammed [Tippu Tip] who ruined my trip – then your property will be seized;

and he added, ‘when you’ve said that, well, leave things to me’.^84^

Tippu Tip’s reasoning, which he attributed also to his men, was that they were obliged to support the Sultan’s decisions. If they didn’t, they would not only go against Islamic injunctions, but also risked being punished (‘your property will be seized’), as indeed happened when Tippu Tip was briefly imprisoned in Zanzibar after helping Livingstone.^85^ On this occasion, although the Sultan was not seriously angry with Tippu Tip (or he would not have softened Tippu Tip’s imprisonment as he did), he had to be seen to concur with the British Consul. Surely, Tippu Tip’s help to Europeans could be turned to his personal advantage. But the collaborative attitudes of both the Sultan and Tippu Tip were primarily intended to salvage the Sultanate and what the Sultanate stood for: the norms that regulated their lives and gave them power.^86^

Tippu Tip had been told by a reliable source (Taria Topan) that Sultan Barghash wanted to make him Wali of Tabora (that is, the Sultan’s ambassador in Tabora) when ‘a Belgian’ (Mr B.) offered Tippu Tip a deal to organize an expedition together. The Belgian would provide guns and powder; Tippu Tip would be responsible for the men. Tippu Tip replied: ‘I am a subject of the Sultan, Seyyid Barghash, and the country of Manyema over which I rule, both it and I are under the authority of Seyyid. I can do nothing without his sanction’. And reiterated: ‘I can do nothing except on the authority of my ruler’.^87^ Following this conversation, Tippu Tip met Seyyid Barghash. He informed the Sultan of Mr B.’s proposition, and the Sultan told him that though he had intended to give him the Wali-ate of Tabora, he now thought it best that he set off again for Manyema. The Sultan exhorted Tippu Tip to meet the requests of Europeans. On another occasion Seyyid Barghash summoned Tippu Tip to meet him in Zanzibar urgently. The Sultan expressed his anxiety:

The Europeans here in Zanzibar want to steal from me. Will it be the hinterland? Those who are dead, who see nothing of this, are at peace. You too are a stranger to it. You will see what is involved.

And Tippu Tip commented, ‘When I heard Seyyid’s words I knew that it was all up’.^88^

Was the Sultan thinking of his ‘peacefully dead’ ancestors’ imperialism in East Africa when he told Tippu Tip that he understood what was involved in Europe’s activities? Or was he referring to his familiarity with European politics, which he witnessed through participation in the main international congresses of the times? Be it as it may, Tippu Tip’s account shows that his own power emanated from Zanzibar’s Sultanate and that both the Sultan and Tippu Tip knew they were facing an unprecedented existential threat. At the end of the 1880s the Sultan’s authority was co-opted by Leopold II for the administration of the Congo Free State. About three months after this conversation between Tippu Tip and the Sultan, Stanley and ten Europeans met Tippu Tip at the Consul’s place to inform them that they wanted Tippu Tip firstly ‘to be Wali of the Belgian area, and your men in these areas to raise the Belgian flag. Secondly, we want you to give us men to [find] Emin Pasha’.^89^ Tippu Tip replied that he had to consult Sultan Barghash,

since both we ourselves and our areas were under his authority. They replied that all authority was in my hands, and that the areas of Manyema were under my control; that I was, in fact, the chief. They wrote down a contract and Mr. H. read it to me. I took it to Seyyid and told him all that had passed. Seyyid advised me to go and to go as far as wanted.^90^

Tippu Tip was given Congo Free State flags to plant in Stanley Falls.^91^ He argued in his memoirs that he recommended to Rumaliza to follow his example and support Europeans rather than act against them:

I advised him to comply with the Europeans in so far as they wanted. Because our power lay in our Sultan. *The extent of my authority – both I, the country and the people – was in his power. He was following the commands of the European more than we were*.^92^

Clearly, Tippu Tip, the Sultan, and their networks felt the growing threat posed by European presence, which was justified in abolitionist terms. Yet, ‘Europeans’ were hardly unified. French, British and Belgians – diplomats, missionaries, and military men – held different views on the trustworthiness of locals and what the next steps should be.

In a letter to Cardinal Lavigerie in the Fall of 1890, Joubert stated that he thought it ‘beyond doubt’ that Rumaliza had received from Said Barghash the mission to subjugate the lake’s shores at the same time that Tippu Tip was tasked with opposing the establishment of Europeans in the Congo Free State.^93^ Joubert also suspected that the British consulate secretly supported Rumaliza and Tippu Tip as their opposition to Belgian expansion benefited ‘*la politique anglaise*’. He reported that Tippu Tip had brought loads of Congo flags and claimed that he had been charged with planting them everywhere. Joubert feared that if indeed such powers had been entrusted to Tippu Tip, ‘the people of Tippu Tip will quietly continue to practice the slave trade in the shadow of the Congo flag as they have been doing up until now in the whole Manyema and beyond’. Joubert was enraged. He was not only intensely hostile to Tippu Tip and his network, but also felt personally exposed:

As for me, I do not understand my position anymore. Tippu Tip said that he would send me flags and if I accept them, it will be fine, but if I refuse, he’ll report [my refusal] to the Belgian consul. *One thing is certain: I shall never accept from him either flag or orders.* Anyway, I have received no official communication that he had jurisdiction over this country. And should the Congo government give him such jurisdiction, *it won’t be possible for me to take orders from the first of all slavers*.^94^

## Controlling and Coercing People

Joubert and Tippu Tip struggled to control labour constantly. For Tippu Tip slavery was also, though not only, a solution to the labour problem, the central element of his commercial prominence. For Joubert, slavery was the reason for his being in East Africa and, yet, combating slavery required enrolling workers and dependents in the new regime of the missionaries.^95^ Understanding Joubert and Tippu Tip’s relation to labour and their respective labour control strategies is therefore key to discerning the causes of their antagonism, which culminated into open conflict. At the end of the century in Tanganyika, controlling people became both the *casus belli* and the crucial means to overcome adversaries.

Trade and travel were labour intensive. Porters were an essential category of workers in long-distance expeditions and thus had bargaining power. Expedition leaders depended on porters, who were usually freemen, though slaves were also frequently used for porterage.^96^ Tippu Tip’s success was partly due to his ruthlessness. He once arranged with his brother a trade expedition at a time of famine.^97^ He managed with difficulty to recruit 700 porters in Zanzibar. Before departure, he distributed food for the six days ahead, expecting that food would be scarce on the road. But when the day of departure arrived, the porters, who were scattered in several villages where they set down the loads, had deserted. Infuriated, Tippu Tip set-off with few trusted servants and enrolled 80 men whom he armed with guns. With this newly constituted group, he reached the villages of the porters, seized their elders and kinsmen, about 200 of them, and bound them up. He then (re-)captured 800 porters and put them in chains.^98^

Success depended on mobilizing social capital in the first place. People could be made to feel obliged in a variety of ways: by mobilizing ties of kinship, religion or other forms of allegiance; by indebting them materially or morally; or by chaining them up and threatening violence against them. In this case Tippu Tip resorted to violence. He took hostage the porters’ elders and relatives, thereby ensuring that the porters would not escape and leave their family at his mercy. Then, by using his superior gunpower, he captured 800 men, who likely included some of the porters who had deserted, put them in chains and reconstituted a large caravan. What sense to make of this episode? Initially, Tippu Tip and his brother were the offended party, who risked losing everything due to a mutiny of porters who had already been fed and partially paid for their future services. But thanks to Tippu Tip’s immediate reaction, they were able to carry out their expedition as planned. This involved enchaining people to force them to work for his caravan during a famine. Chains prevented escape, the easiest form of resistance in this environment. Paradoxically, the famous abolitionist Verney Lovett Cameron wrote apologetically about Tippu Tip’s habit to put slaves in chains:

The only drawback I experienced to the comfort of Tipo-tipo’s camp was the number of slaves in chains who met my eyes at every turn; but, except being deprived of their freedom, and confined in order to prevent their running away, they had a tolerably easy life, and were well fed.^99^

Compassion for the enslaved was not one of the many qualities that Tippu Tip claimed for himself. He often bragged about his firmness toward enslaved persons and didn’t show pity for the predicaments that may have triggered their allegedly impulsive reactions: hunger, exhaustion, fear, pain for the loss of loved ones. Trusted slaves often fought with and for slaveowners. Their knowledge of the territory and of events they may have witnessed was valued and they were consulted alongside free dependents.^100^ But Tippu Tip also blamed slaves easily. The story of a slave boy who sought revenge against a man from an enemy group who had split his millet is a case in point. Tippu Tip told the slave boy that a rash reaction against these enemies could have serious consequences for their whole party. He ordered the boy to calm down and promised to give him back the equivalent of his lost millet. But the boy did not pay heed to Tippu Tip’s admonitions and went to the enemy armed with a gun. Tippu Tip called him back and hit him, but a crowd of people, having seen the boy armed, turned against Tippu Tip’s group.^101^ A fight ensued. Tippu Tip tried to persuade the enemy chief to call his men off, but the chief didn’t, and Tippu Tip had him killed. Upon seeing their chief shot, locals dispersed. Although it seems unlikely that a slave’s boldness could occasion an uprising against Tippu Tip, in this passage Tippu Tip blames the boy’s mindless resentment for a trivial incident for provoking events that led to the death of many, including an enslaved girl married to Tippu Tip’s personal slave Bangura (‘lion’). On seeing his wife killed, Bangura said: ‘When it comes to a fight, I shall be the first to die!’ About one week later, conflict broke out between Tippu Tip’s party and local groups. Bangura left at the first war-cry; he ‘threw himself at the horde; they speared him to death. By the time we appeared he was already dead’.^102^ Tippu Tip portrays slaves as impulsive and unreflective, a trait connected with alleged inability to govern oneself, a trope in ideologies of slavery that justify the slaveowners’ coercion as necessary.

In Joubert’s moral compass empathy toward the enslaved was not a sign of weakness but a virtue that abolitionism encouraged. Joubert returned to Europe in 1884. In the first stretch of this trip, he joined a caravan that carried ivory and slaves to the coast and witnessed the enormous suffering of traded slaves, which influenced his commitment to fight against the slave trade and slavery. His succinct description of the pitiful conditions of enslaved children in a letter to Cardinal Lavigerie sums up his experience: ‘we march slowly because the slave children, who have been burdened by their masters with their mats, their provisions, and even with small ivory tusks, drop their burdens’.^103^ From mid-November the rains made daily marches even more exhausting. The sufferings of the enslaved filled the pages of his travel journal.^104^ On 22 November 1884, ‘Makanga wants to kill a little Manyema, his slave, who can’t walk anymore. I give him a dose of quinine which gets him back on his feet’. But when they stop to drink at a stream this slave escapes. Lost in the forest of bamboo, he can’t be found, and Joubert comments that he might die of hunger if he is not found by a local who’ll re-enslave him.^105^ On the following day,

a Manyema slave brought by the men of Rumaliza remained behind as he was unable to keep marching. Those who accompanied him had taken away from him the piece of cloth that covered him. So he ran away in the woods, where death awaits him.^106^

Two days later, a porter declared that he couldn’t carry his load anymore. Joubert, unable to find other porters, carried it himself.^107^ Further down the road he found two porters with ‘a little Manyema of 9–10 years’ who has pains to his knees and can’t walk anymore. Unable to carry him, Joubert tried to support him and make him walk, but he couldn’t stand on his feet and was left behind. Then ‘a little Manyema slave girl’ couldn’t carry the package given to her anymore. When it started raining, she couldn’t keep walking, so Joubert carried her. She was feverish and cold like ice; Joubert feared for her life. He estimated that less than a quarter of all enslaved ‘Manyema’ reached the coast.^108^ His party arrived at the White Father’s station in Kipalapala just before Christmas. Six months later Joubert left East Africa.

Back in France, at the end of July 1885 General Charette organized a meeting to celebrate the silver wedding anniversary (25 years) of the Zouave regiment.^109^ Joubert’s African work was celebrated.^110^ Meanwhile, the Belgian officer Emile Storms had established a station in Mpala on the western coast of Lake Tanganyika, acting on behalf of the International African Association (IAA) and the King of Belgium.^111^ The IAA’s official mandate was to expand scientific knowledge on the region, assist European travellers, and eradicate slavery.^112^ But it also served the purpose of consolidating Leopold II’s control over the Congo basin.^113^ The General Act of the Berlin Conference of 1884–1885 vowed to abolish the slave trade in articles 6 and 9. At the Conference Leopold II ceded Mpala (till then an IAA outpost) to the White Fathers as part of a compromise between Belgium and Germany.^114^ Storms officially gave the keys of the two stations of Mpala and Karema to the missionaries, who settled in the stations on 26 July 1885.^115^ Fathers Isaac Moinet and Auguste Moncet had made theirs the dream of Cardinal Lavigerie to create a Christian Kingdom in the heart of Africa.^116^ They thought that Mpala could serve this purpose and that Joubert could act as the first Christian king.^117^ This dream would never be realized, but Father Moinet asked Cardinal Lavigerie to call Joubert back. Lavigerie contacted Charette who informed Joubert who returned to Tanganyika.

In May 1886 Joubert started his trip and in December 1886 he reached Karema. After two months with the Fathers, he moved to Mpala on 20 March 1887. He had an informal mandate that gave him broad powers. He kept a journal in which he recorded how he implemented his administrative duties. The villages of the White Fathers grew mainly through the integration of enslaved persons who were ransomed by the fathers or fugitives who sought refuge with them. For example, Karema acquired about 2000 ransomed slaves between 1889 and 1900.^118^ Other slaves were donated as gifts by chiefs from surrounding villages as a gesture of good will or to obtain other gifts they valued in return (such as cloth). These villages also grew when requests by free chiefs to settle with their people on lands controlled by the Fathers were accepted. In the late 1880s Joubert’s

Christian Kingdom could exist as a *de facto* colony or state because the missionaries constituted the only effective European presence along the western shores of the lake. […] The Captain raised a force of loyalists and threatened counter-attack if tributes were not brought manifesting subservience to him.^119^

Beyond defending allies, opposing foes, and overseeing military operations, Joubert entertained diplomatic relations, minutely documented in his diaries, that involved the exchange of presents with local chiefs.^120^ Chiefs often gave slaves. Sealing an alliance involved chiefs committing personally and formally to accept Joubert’s rules: ‘people of Tamwa bring a child and say they want to become our people. […] We accept on condition that Tamwa comes in person to make his request’.^121^ Upon joining, allies were given a flag to plant in their village, which displayed publicly their subjection to the Fathers and Joubert:

I give the flag to the people of Kaseba. Kisavi, who complains about Mweru, wants to resettle in his old country near Kazeba on the Lufunze [*sic*]. Authorisation is given, but if he wants to be our man he must come in person to accept our conditions and, when he does, he’ll receive the flag.^122^

After a series of violent accidents, on 17 May 1887 Joubert circulated a Law on the Killing and Sale of Slaves.^123^ This law included death penalty for the most serious offences, caning, and fines.^124^ Tensions arose between Joubert and Father Coulbois, Pro-Vicaire of the Haut-Congo stationed in Kibanga. The latter (who elsewhere had expressed the view that slavers should be hung or shot dead^125^) thought that the former was exceeding his mandate: ‘he believes himself a king and acts accordingly by establishing regulations indiscriminately’.^126^ Coulbois wanted to abolish Joubert’s law.^127^ Joubert complained with Lavigerie, who sent a document entitled ‘Rules on the functions of Captain Joubert’ which confirmed the broad remit of the powers that the Cardinal saw fit to delegate to the Captain.^128^ Almost every day locals came to Joubert, who adjudicated trials almost exclusively on contested rights in persons.

Kasabala is accused of having killed one of his slaves and a woman he took from Rutuku. His slave apparently sold or helped escape two women. He allegedly killed the woman of Rutuku as revenge. He is condemned to pay three slaves to the Government. If he pays grown-up men or nubile women, he’ll give two.^129^

Ngombi, our blacksmith, claims his mother back from Fongo who keeps her as a slave after having already taken two slaves and two pickaxes from him for his brother, and his sister whom he was detaining without cause.^130^

Mtono’s father says that Kisakulo is claiming his son as a slave, because his son, a child, had told Kisakulo that he would give him a young slave for a gun. […] The father would like to give a young slave, and is asking for a gun. I prohibit him to do so and I tell him to answer Kisakulo that if he takes people from him I will beat him.^131^

Katambwa’s man comes to complain that a woman from Kisavi’s group had his wife and three other people taken from him to sell them to Makutubu … ^132^

Customary law had resorted to penal slavery as a punishment. Joubert turned this logic on its head and punished culprits by imposing penal manumission. This involved forcing them to free their slaves and transfer their rights in the latter to Joubert, who thereby achieved manumissions without incurring the cost of paying ransoms. Once liberated, formerly enslaved persons feared the potential backlash of their former masters. They often ended up joining the missionaries’ settlements. People changed hands and shifted across different regimes of rights in persons.^133^ These regimes were incompatible, not least because the one supported by Europeans was predicated on the suppression of the others.

## Escalation of Violence

By the end of the decade, European imperialism was rampant. Lavigerie exalted the humanitarian ideals of the King of Belgium Leopold II, but also acknowledged the enormous obstacles faced by Belgium’s Catholic suzerain. In a report to the Pope, Lavigerie noted that the King’s visions were not proportionate to the means at his disposal.^134^ The Congo Free State, which he was mandated to rule upon by the European powers, was ten times the size of Belgium. Leopold II had placed the Lower Congo (closer to the Atlantic) under the control of Belgian administrators; Lavigerie saw these as ‘free thinkers’ whose immorality and corruption were ruinous to the country. Then, in order not to appear to abandon completely the northern half of the country, the Upper Congo, the King placed it under the authority of ‘Muslim slavers, on top of whom there is *the most famous brigand of all Africa, called Tipo-Tipo. And it is this that led to the appalling devastation of these regions*’.^135^ After the Congo Free State, the Cardinal continued, the worst region in terms of the gravity and magnitude of the slave trade was Zanzibar, which the Cardinal connected to Zanzibar’s previous Sultan Barghash’s bestowal of power on the Upper Congo to ‘Muslim slavers’ (including Tippu Tip). The Cardinal’s logic, which exonerated Leopold II and placed the blame squarely at the feet of Barghash and Tippu Tip, fuelled European violence.

Joubert’s position led to confrontations with locals opposed to what they saw as his interference in their country and mores. He sustained multiple attacks, which he faced with the network of local chiefs who had become his allies. The main threat to Joubert (who had moved twice, first to Lisonge with his wife and then again to a new village he founded and named St-Louis-de-Mrumbi) was posed by the hostility of the powerful ‘Arab’ Rumaliza. The latter wrote to Father Coulbois. His letter reveals his and his allies’ views on Europeans and on Joubert specifically. Written in poor French by a local translator, it is filled with accusations and direct threats: ‘you have weakened my entire country’ and ‘I am not at all happy’, Rumaliza wrote. He targeted specifically Captain Joubert:

I do not want the Captain to remain in my country … the Captain is not a priest and all the Arabs want to kill him … [The Sultan of Zanzibar] forbids me to kill you [and so] today you have made war [against me], you have taken all my affairs, all the Arabs of Ujiji complain to Rumaliza and ask him permission to kill you, and you, Father Coulbois, if you are under my protection, you must reply … every day you do things without asking my permission, [but] you are not in your country … If you want to be my friend, give me back my affairs, if you don’t, I will chase you away from my country, and write to the Captain today … a second time, I do not want him to remain there.^136^

Joubert suspected that Rumaliza was hoping to acquire a position analogous to that of Tippu Tip for the Congo Free State.^137^ Tensions turned into open conflict. Rumaliza’s first attack against the Captain failed because Rumaliza’s boat was destroyed by a storm: all those around Joubert saw this as intervention of the divine providence. But this was not the end of Rumaliza’s offensive. French and Belgian actors mobilized to defend their interests in the region. General Charrette sat on the board of directors of France’s anti-slavery association. He started collecting funds and recruiting volunteers to support Joubert.^138^ But diplomatic tensions between European empires made it impossible for a French expedition to happen. In the end, the Belgian Antislavery Committee supported the organization of the ‘Joubert relief expedition’ commanded by a Belgian. Captain Jules Alphonse Baron Jacques de Dixmude, commonly referred to as Captain Jacques, was selected for this task.^139^ When he met Joubert, Captain Jacques naturalized him as a subject of the Congo Free State, as Joubert would have raised suspicion had he retained only his French nationality while commanding military operations in the Congo Free State.^140^ The French Fathers, too, had to be replaced by Belgian missionaries of the same religious order.^141^

Rising competition over control of the east African coastal region and its hinterland territories up to Lake Tanganyika and in the Congo Basin and Katanga soured relations between the representatives of competing European empires as well as between Europeans and ‘Arab’ elites.^142^ Violence escalated.^143^ The travel journal published by the brother of Alexis Vrihoff, young member of the expedition who died ‘heroically’ fighting under the command of Jacques and Joubert, tells of how Belgian officers presented the head of an ‘Arab’ slaver as trophy to Joubert.^144^ There is no mention of this episode in Joubert’s diary: was Joubert too ashamed to report the fact, or did this fact never happen? Vrithoff himself died shortly afterwards.^145^ The arrival of Alexandre Delcommune, who was returning to Europe after his Katanga Exploration Expedition (1890–1893), brought reinforcements to Jacques and Joubert. The Expedition had mixed political and economic ends and was Belgium’s response to Cecil Rhodes’ aim to unite British African possessions from South to North (1890–1893).^146^ It had started in Matadi, the main Atlantic port of Kongo, and had moved in a west–east direction. It included few European officers and a militia of over 100 ‘Hausa’ soldiers recruited in Lagos, each of whom was equipped with personal baggage, weapons and munitions.^147^ Delcommune and his Hausa troops arrived on the shore of Tanganyika in August 1892.^148^ Joubert explained Jacques’ situation and Delcommune said that he would see if his Hausa men would agree to go to Albertville, where Jacques was under siege and in a critical position.^149^ About twenty Hausa joined in for some extra pay.^150^ But the majority of the Hausa refused to march against the men of Rumaliza whom they saw as fellow Muslims.^151^

The Hausa of Delcommune were neither the only West Africans nor the only Hausa in Tanganyika. And not all West Africans, not even Hausa amongst them, sided with Muslims. If ethnicity never determined allegiance anywhere, this is particularly true for those who came from slavery who had experienced discrimination and abuse within their own communities, often in spite of common ethnic and religious background. The experience of enslavement had nourished a distinctive consciousness among communities of African Christian converts. These men and women interacted on a daily basis both with Europeans and with other African groups, but it would be wrong to conflate them with these other actors. The few writings we have by them attest to distinctive values and moral judgements. Their actions as portrayed in mission diaries and correspondence show that whatever else they did, they worked against slavery and sustained the enslaved.^152^

Joseph Gatchi, Hausa of Kano, and Adrien Atiman, Songhay of Tindirma, were West Africans enslaved as children, liberated by the White Fathers, and trained as doctors in Malta.^153^ They never forgot their West African origins.^154^ They were sent as doctor-catechists to Tanganyika with the White Fathers’ caravan of 1888. In 1889 Gatchi was assigned to the post of Kibanga, where he worked until the post was suppressed in 1893. He seconded Captain Joubert in Mpala and in St Louis-de-Mroumbi. In June 1894 Captain Descamps asked the Apostolic vicarate whether Gatchi could replace the Commander of the post of Moliro. The apostolic vicar agreed, and from 15 July 1894 Gatchi found himself temporarily assuring the safety of the region south of Lake Tanganyika. He returned to St Louis de Mroumbi in September 1894 and remained there until 1905, when an epidemic of trypanosomiasis rendered necessary the resettlement of the station. Committed to his work for the mission as doctor and catechist, he refused to return to Moliro.^155^ Every day Gatchi cured and taught Christian religion and music to orphans who, like him, had been enslaved and liberated. He supported the military operations against Rumaliza and tragically lost his first wife in childbirth when he decided to part from her to cure Captain Jacques engaged in military operations against Rumaliza.^156^ His commitment to the abolitionist cause, if not necessarily to Jacques, was unconditional.

Adrien Atiman worked in Karema from 1889 until his death in 1956. Most of Atiman’s writings, and his autobiography, focus on two themes: conversion to Christianity and his work as a doctor. He was purchased from slavers by Father Deguerry and wrote to him regularly: ‘I shall never forget you, very reverend father, liberator of [my] body and of [my] soul … ’^157^ In a letter of New Year wishes to Lavigerie, Atiman wrote:

May the Good God let you touch the heart of the civilized world through the mission that the Holy Father Pope Leo XIII assigned to you in favour of so many unfortunate slaves who are still in irons, in cangues, naked, abandoned in their final hour. It is not long ago that we saw many slave caravans in this state going to the coast.^158^

Gatchi, Atiman and other African liberated slaves shared many of the aims of the missionaries, were committed abolitionists, and equally anti-Arab.^159^ But their main commitment was to their God, to the enslaved, and to those who cared for the enslaved. Atiman praised the White Sisters’ support to African women and slaves:

What an immense good have the sisters in Karema done in both the spiritual and temporal domain. I – deep-black negro [*nègre bien noir*] with frizzy hair – I tell you that the sisters arrived on time to collect what remained of the slave women and girls around Karema to turn them into Christians, angels for heaven and family mothers, and now their old masters and mistresses are happy to come to them, and their children are the godchildren of the children of their former slaves whom they now call their brothers, their sisters, brothers in law, their sisters in law!!! What I am telling you are no hyperboles, no turns of phrase, they are the truth. The black woman feels free, feels like a person, not a simple object of trade.^160^

In the long term, Islamic rule would be confronted less with challenges posed by the likes of Delcommune, Jacques, and Joubert, than with the distinct worldviews of communities of Christian ex-slaves who, having been nurtured by tens, possibly hundreds, of men like Gatchi and Atiman, rejected ideologies that tolerated slavery.

## Conclusions and Caveats

The imperialist antislavery of Europe provoked exceptional violence, a violence at once material and ideological. This article is an attempt to describe how this happened and analyse the local implications of this process. Sayyida Salme bint Said ibn Sultan, Princess of Oman and Zanzibar, was born in Zanzibar in 1844. She converted to Christianity and was baptized ‘Emily’ in 1866, married the German Rudolph Heinrich Ruete, and moved to Germany.^161^ Her memoirs, published in 1886, contain the reflections of a woman who navigated across the worlds of Joubert and Tippu Tip.^162^ Having discussed the hypocrisy and inhumanity of certain European abolitionists, she commented:

Nobody should be surprised if the Arabs, after such experiences, are filled with the greatest suspicion against Europeans; they wish for those happy times, when they were still unexposed to the latters’ all-subverting ideas. The abolishment of slavery, so the Arabs think, is only pursued with the aim to ruin them and thus to harm Islam.^163^

Considering the causes of ‘Muslim fanaticism’, she reasoned that what occasioned it was ‘the urge to self-preservation; the stronger this urge asserts itself with them, the more they are threatened in *their most vital interests* by reckless, ignorant and often unworthy representatives of civilization and Christianity’.^164^ Sayyida Salme/Emily Ruete’s thoughts corroborate this article’s argument. Although they stood against each other, Leopold Joubert and Tippu Tip had more in common than it may at first appear. They were both opposed to the new logics that threatened *their most vital interests* and the authority of the rulers they served.

Ultimately, they fought losing battles: the political and temporal power of both the Pope and the Sultan of Zanzibar shrank substantially during their lifetimes. Virtually the entire continent of Africa (except for Liberia and Ethiopia) was occupied by Europe’s empires shortly after these events. By the end of the nineteenth century, the moral force of abolitionism was such that a self-arrogated mission to end slavery worldwide could be used to legitimise European interventionism. This, as Princess Salme noted, was evident to ‘Arabs’ convinced that slavery’s abolition ‘was only pursued with the aim to ruin them *and thus to harm Islam*’. There were differences of vision and attitude between Tippu Tip and Rumaliza, between Joubert and Jacques. But as actors identified with institutions they saw as menaced, a logic of crisis required them to abandon all hesitation, nuance, and self-criticism. People took sides with a broadly defined Self and against a demonized Other in the name of cultural-religious values the preservation of which was thought to justify violence. This entailed suspending critical engagement with these values and overlooking the ‘differences within’ one’s networks. Perhaps paradoxically, the perceived necessity to stop the violence of slavery at a time when commitment to antislavery could contribute to restore the Pope’s moral capital made men like Joubert complicit with the violence of colonial aggression, which other men like Alexandre Delcommune and Jacques de Dixmude were undoubtedly more attuned with. Faced with European brutality and hypocrisy, rising numbers of ‘Arabs’ joined Rumaliza’s resistance. Those most concerned with the suffering of the enslaved, former slaves like Atiman and Gatchi, acted quietly in support of victims, both because they were forced into subordinate positions in increasingly racist organizations and because, as European violence and racism escalated, they must have doubted the virtue of their superiors.

When abolitionism fuelled movements that sought to eradicate the harshest form of exploitation, it sustained historical virtuous circles of human emancipation. But made to serve imperialist ends, it legitimized vicious circles of self-legitimising violence, as perpetrators on all sides thought they occupied a position of moral and civilisational superiority. As they sought to salvage doomed institutions, Joubert and Tippu Tip felt entitled to kill and coerce. This is what happened on the shores of Lake Tanganyika at the end of the nineteenth century. But if this was the story of a vicious circle, it was not a dead end. It was also a beginning. In these events a process of emancipation unfolded, in which thousands of liberated slaves cultivated new ideals. They were moved by an intense aversion toward rationales that had justified the oppression of persons construed as inferior, enslavable, and coercible at will. Integrated in European missionary institutions, formerly enslaved persons continued to face exploitation.^165^ Their labour was extracted through coercion and, following colonial occupation, they were forcibly recruited into state service and, in Eastern Congo, the *Force Publique*.^166^ Although life continued to be difficult, the formerly enslaved and their descendants took advantage of the opportunities that had become available to them to resist oppression. Many of those who had experienced slavery used colonial institutions not only to free themselves, but also to support others who were still struggling to self-emancipate.^167^ They developed a rhizomatic abolitionism that is only just beginning to be explored. This article prefigures these developments, as new subjectivities and solidarities were formed in these dynamics.

It is often stated that abolitionism was instrumentalised as a discourse that legitimized Europe’s rising imperialism and the so-called ‘scramble’ for Africa. But this history was not solely made by Europe. The attitudes of different African actors toward slavery and abolition were constitutive of these global historical processes.^168^ This article provides a microhistory of interconnected strategies unfolded as a consequence of a certain problematisation of African slavery.^169^ It delineates the individual experiences that shaped, and were conditioned by, religious proselytism, abolitionism, imperialism, colonialism and anticolonial resistance. It argues that contingency matters. The sense of urgency that evolved simultaneously in the separate moral worlds of Joubert and Tippu Tip created a momentum in which institutions and social networks promoted an escalation of violence.^170^ It may be argued that both Leopold Joubert and Tippu Tip were, in different ways, exceptional and that it is difficult to generalize based on their individual trajectories. If anything, they were ‘normal exceptional’ cases (*l’eccezionale normale*).^171^ They strove to reproduce moral outlooks that were broadly shared by people who felt collectively threatened. The interventionist abolitionism that developed in the mid-1800s was ‘normal’, too, and had become a mantra in international politics. The question of what to do about African slavery is the story-line connecting the multiple sites, worldviews, institutions, and networks examined here.^172^ (Anti-)abolitionism, thus, functions like a thread (*filo conduttore*) for us to follow as we seek to make sense of the judgements of value that directed people’s actions and shaped their lived experience (*scienza del vissuto*).^173^ Such judgements supported the ways of life of persons who thought they were following divine laws. They permeated fanaticism camouflaged as God’s will.

The article’s inquiry has encountered a major difficulty emanating from the fact that the words we use are morally coloured and normative today, as they were in the past. They are at once analytical concepts and judgements of value. Terms such as ‘slave’, ‘slavery’, ‘abolitionism’, ‘colonialism’, and ‘anticolonial resistance’ are tied up with identity in ways that change in time and across political positions. Our own involvement in this history, our position in relation to its struggles, complicates our interpretations. To Leopold Joubert and Tippu Tip the violence they unleashed was about what they thought God wanted them to do; their role in relation to people they recognized as authorities (sultans, popes, kings); their sense of impending catastrophe in the face of new rationales that challenged their existential worlds. Surely, Joubert also happened to be the carrier of a brutal imperialism; Tippu Tip was a slave trader who reproduced a system of slavery and caused intense pain to the persons chained-up in his caravans. But by focusing on the complexity of their predicaments and on how they justified their choices to themselves and to their contemporaries, we can discern the mechanisms that set major historical transformations in motion with dramatic consequences. Our present concerns are not relevant to making sense of their actions. Although our ‘truths’ should not influence our historical analysis, critical engagement with the ‘truths’ of others who lived in the past should make us reflect on how new problematisations and politicisations of slavery affect present-day contingencies.

## Notes

1 For a parallel argument in the Atlantic context, see Christopher Leslie Brown, *Moral Capital: Foundations of British Abolitionism* (Chapel Hill: University of North Carolina Press, 2006), and Seymour Drescher, *Abolition: A History of Slavery and Antislavery* (New York: Cambridge University Press, 2009), 205–41. See also Amalia Ribi Forclaz, *Humanitarian Imperialism: The Politics of Anti-Slavery Activism, 1880–1940* (Oxford: Oxford University Press, 2015). On the region discussed in this article, see Raphaël Chériau, *Imperial Powers and Humanitarian Interventions: The Zanzibar Sultanate, Britain, and France in the Indian Ocean, 1862–1905* (London: Routledge, 2021).

2 Mary Douglas, *How Institutions Think* (London: Routledge, 1987), 4.

3 John Fabian Witt, ‘Two Conceptions of Suffering in War’, in *Knowing the Suffering of Others: Legal Perspectives on Pain and Its Meanings*, ed. Austin Sarat (Tuscaloosa: University of Alabama Press, 2014), 133.

4 The Battle of Solferino was the decisive confrontation in the Second Italian War of Independence: Napoleon III and Victor Emmanuel II sided against Austrian Emperor Franz Joseph I. It also was the last major battle in which all armies were commanded by their monarchs.

5 For an analysis of Dunant’s thinking in relation to the development of international policies for the protection of war victims and wounded soldiers, see Andre Durand, ‘The Development of the Idea of Peace in the Thinking of Henry Dunant’, *International Review of the Red Cross* 250 (1986): 16–51. A more critical reading of Dunant’s ideas has been advanced by Doreen Lustig and Eyal Benvenisti, ‘Revisiting the Memory of Solferino: Knowledge Production and the Laws of War’, in *International Law’s Invisible Frames: Social Cognition and Knowledge Production in International Legal Processes*, ed. Andrea Bianchi and Moshe Hirsch (Oxford: Oxford University Press, 2021; online edn, Oxford Academic, December 23, 2021), https://doi-org.libproxy.ucl.ac.uk/10.1093/oso/9780192847539.001.0001 (accessed April 22, 2024).

6 James Turner Johnson compares Christian just war and Islamic jihad traditions in *Ethics and the Use of Force: Just War in Historical Perspective* (London: Routledge, 2011).

7 https://www.vatican.va/content/pius-ix/it/documents/encyclica-quanta-cura-8-decembris-1864.html. See also Ernesto Rossi, *Il Sillabo e dopo* (Milan: Kaos, 2000), based on Ernesto Rossi’s original work published in the late 1950s; E.E.Y. Hales, *The Catholic Church in the Modern World: A Survey from the French Revolution to the Present* (New York: Hanover House, 1958).

8 On how multiple positionalities complicate polarities such as colonisers and colonised, or abolitionists and slavers, see Camille Lefebvre and M’hamed Oualdi, ‘Remettre le colonial à sa place’, *Annales. Histoire, Sciences Sociales* 72, no. 4 (2017): 942; Frederick Cooper, *Colonialism in Question* (Berkeley: University of California Press, 2005), 3–32.

9 Benedetta Rossi, ‘Global Abolitionist Movements’, *Oxford Research Encyclopedia of African History*. Published online on July 19, 2023, https://doi.org/10.1093/acrefore/9780190277734.013.945 (accessed April 22, 2024).

10 David Livingstone (ed. Horace Waller), *The Last Journals of David Livingstone, in Central Africa, from 1865 to His Death: Continued by a Narrative of His Last Moments and Sufferings, Obtained from His Faithful Servants Chuma and Susi* (London: John Murray, 1874).

11 https://www.vatican.va/content/leo-xiii/en/encyclicals/documents/hf_l-xiii_enc_20111890_catholicae-ecclesiae.html. This had been preceded by two other encyclicals that also addressed the issue of slavery: *In plurimis*, on slavery’s abolition, sent to the bishops of Brazil on May 5, 1888, https://www.vatican.va/content/leo-xiii/en/encyclicals/documents/hf_l-xiii_enc_05051888_in-plurimis.html, and *Libertas*, on the nature of freedom, June 20, 1888, https://www.vatican.va/content/leo-xiii/en/encyclicals/documents/hf_l-xiii_enc_20061888_libertas.html.

12 Sacra Congregazione degli Affari Ecclesiastici Straordinari. *Abbozzo di enciclica sulla schiavitu*, n.d., Archivio Apostolico Vaticano (hereafter AAV), Segr. Stato, Spoglio Leone XIII, b. 82, fasc. 392, ff. 7–8. See also Karl Josef von Hefele, ‘Esclavage’, in *Dictionnaire encyclopédique de la théologie catholique*, ed. Joseph Wetzer and Benedikt Welte, trans. I. Goschler (Paris: Nabu, 1858), tome VIII, 31–41.

13 For a general study, see Pius Onyemechi Adiele, *The Popes, the Catholic Church, and the Transatlantic Enslavement of Black Africans 1418–1839* (Hildesheim: Georg Olms Verlag, 2017), see especially 405–21.

14 On Catholic abolitionism in Africa in this period see Lorenzo Ettorre, ‘La società antischiavista d’Italia (1888–1937)’, *Studi Storici* 53, no. 3 (2012): 697–720; Gabriele Montalbano, ‘Italian Abolitionism in Late and Post-Ottoman Libya (1890–1928)’, *Afriche e Orienti* 23, no. 3 (2021): 204–21; Giacomo Ghedini, *Da ‘selvaggi’ a ‘moretti’: schiavitù, riscatti e missioni tra Africa ed Europa, 1824–1896* (Firenze: Firenze University Press, 2023).

15 The Italian historiography on this theme is broad. See Giovanni Spadolini, *L’opposizione cattolica da Porta Pia al ’98* (Firenze: Vallecchi, 1954). For a review of the main arguments, see Ulderico Parente, ‘Il Risorgimento e il paradigma intransigente’, *Enciclopedia Treccani*, published online at: https://www.treccani.it/enciclopedia/il-risorgimento-e-il-paradigma-intransigente_(Cristiani-d'Italia)/ (accessed April 22, 2024).

16 Charles Lavigerie, *Memoir secret addressé à la Sacrée Congrégation de la Propagande sur la création de Vicariats Apostoliques dans l’Afrique Equatoriale*, January 2, 1878, General Archive of the Missionaries of Africa (hereafter GAMAfr), Lav I, 39, III–1.

17 For example, Lavigerie’s London speech was published as an appendix of Alexis M.G.’s book *La Barbarie Africaine et l’action civilisatrice des missions catholiques au Congo* (Liege : Dessain Imprimeur, 1889), which opens its first chapter as follows : ‘Islam (*Le mahométisme*), principal cause of the slave trade … ’ (p. 1). See also Diego Sarrió Cucarella, ‘Lavigerie and Islam: An Ambiguous Legacy’, *Islamochristiana* 46 (2020): 141–56.

18 His lecture on African slavery delivered in St Sulpice on 1 July 1888 was based on his notes from reports by David Livingstone, Samuel Baker, Henry Morton Stanley, Verney Lovett Cameron, Christopher Rigby, Étienne-Félix Berlioux, Gustav Nachtigal, Georg Schweinfurth, and several missionaries based in Africa from his and other religious orders, GAMAfr, Lav A17.4. The lecture was published in the French daily newspaper *Le Monde* on July 18, 1888 and excerpts were published in numerous European newspapers.

19 Letter of Pietro Alasian, Patriarch of Cilicia, to Cardinal Mariano Rampolla, Secretary of State, October 7, 1888, AAV, Segreteria di Stato, Rubriche, anno 1894, rubr. 204, fasc. 1, file 78217.

20 Ahmad Chafiq, *L’esclavage au point de vue musulman* (Le Caire : Ahmad, 1891), 5 (my translation).

21 Those involved at the time were aware of how European accusations were perceived by Islamic rulers, as attested by Samuel Baker’s defence of Egypt’s Khedive against the ‘unsympathetic expressions’ of Europe’s public opinion, see Samuel White Baker, *Ismailïa: A Narrative of the Expedition to Central Africa for the Suppression of the Slave Trade Organized by Ismail, Khedive of Egypt* (London: Herper and Brothers, 1879), preface to the second edition.

22 Abdulaziz Lodhi, *The Institution of Slavery in Zanzibar and Pemba* (Uppsala: Scandinavian Institute of African Studies, 1973). This study is based on local sources and the author’s experience growing-up in Zanzibar.

23 Jan-Georg Deutsch, ‘Notes on the Rise of Slavery and Social Change in Unyamwezi c. 1860–1900’, in *Slavery in the Great Lakes Region of East Africa*, ed. Henri Médard and Shane Doyle (Oxford: Oxford University Press, 2002), 76–110; on north-eastern Congo see David Northrup, ‘The Ending of Slavery in the Eastern Belgian Congo’, in *The End of Slavery in Africa*, ed. Suzanne Miers and Richard Roberts (Madison: University of Wisconsin Press, 1988), 464–67; on East Central Africa see Edward Alpers, *Ivory and Slaves: Changing Pattern of International Trade in East Central Africa to the Later Nineteenth Century* (Berkeley: University of California Press, 2023), 209. Philip Gooding has argued in favour of a more nuanced analysis of dependency and against the polarisation between slavery and freedom, ‘Slavery, “Respectability”, and being “Freeborn” on the Shores of Nineteenth Century Lake Tanganyika’, *Slavery and Abolition* 40, no. 1 (2019): 147–67.

24 Richard Reid, *Warfare in African History* (Cambridge: Cambridge University Press, 2012), 117–20.

25 Stephen Rockel, ‘Caravan Porters of the Nyika: Labour, Culture and Society in Nineteenth-century Tanzania’ (PhD diss., University of Toronto, 1997). Enslaved labour included not just men, but also women and children, see Stephen J. Rockel ‘Enterprising Partners: Caravan Women in Nineteenth Century Tanzania’, *Canadian Journal of African Studies* 34, no. 3 (2000): 748–78.

26 Thomas Vernet, ‘Slave trade and slavery on the Swahili coast (1500–1750)’, in *Slavery, Islam and Diaspora*, ed. Behnaz Mirzai, Ismael Musah Montana, and Paul Lovejoy (Trenton: Africa World Press, 2009), 49–50.

27 Mtoro bin Mwinyi Bakari, *The Customs of the Swahili People: The Desturi Za Waswahili of Mtoro Bin Mwinyi Bakari and Other Swahili Persons*, trans. J.W.T. Allen (Berkeley: University of California Press, 1981), 169–70; Edward A. Alpers, ‘Debt, Pawnship and Slavery in Nineteenth-Century East Africa’, in *Bonded Labor and Debt in the Indian Ocean World*, ed. Gwyn Campbell and Alessandro Stanziani (London: Routledge, 2015), 35–6.

28 Fred Morton, ‘Pawning and Slavery on the Kenya Coast: The Miji Kenda Case’, in *Pawnship, Slavery, and Colonialism in Africa*, Paul Lovejoy and Toyin Falola (Trenton: Africa World Press, 2003), 239.

29 Bernard K. Freamon, ‘Definitions and Conceptions of Slave Ownership in Islamic Law’, in *The Legal Understanding of Slavery: From the Historical to the Contemporary*, ed. Jean Allain (Oxford: Oxford University Press, 2012), 144; William G. Clarence-Smith, ‘Debt and the Coercion of Labour in the Islamic Legal Tradition’, in *Bonded Labour and Debt*, ed. Campbell and Stanziani, 21–30.

30 The Shafi‘i school was perhaps more influential in the earlier periods, with Shi‘a and Ibadhi actors rising in influence since the eighteenth century as trade and legal operators. See Randall L. Pouwells, *Horn and Crescent: Cultural Change and Traditional Islam on the East African Coast, 800–1900* (Cambridge: Cambridge University Press, 1987); Omar H. Ali, *Islam in the Indian Ocean World: A Brief History with Documents* (Boston: Bedford St. Martin’s Press, 2016); Sebastian Prange, *Monsoon Islam: Trade and Faith on the Medieval Malabar Coast* (Cambridge: Cambridge University Press, 2018).

31 Fahad Bishara, *A Sea of Debt: Law and economic life in the Western Indian Ocean, 1780–1950* (Cambridge: Cambridge University Press, 2017).

32 Hollian Wint, ‘Financing the Indian Ocean Slave Trade’, *Oxford Research Encyclopedia of African History*, published online 15 September 2022, https://doi.org/10.1093/acrefore/9780190277734.013.907 (accessed April 22, 2024).

33 Carl Velten, *Swahili Prose Texts: A Selection from the Material Collected by Carl Velten from 1893 to 1896*, trans. Lyndon Harries (London: Oxford University Press, 1965), 79.

34 Lodhi, *The Institution of Slavery*, 14–15.

35 Elisabeth McMahon, ‘Trafficking and Re-enslavement: Social Vulnerability of Women and Children in nineteenth century East Africa’, in *Trafficking in Slavery’s Wake: Law and the Experience of Women and Children in Africa*, ed. Richard Roberts and Benjamin Lawrance (Athens: Ohio University Press, 2012), 29–44; Edward Alpers, ‘The Story of Swema: Female Vulnerability in Nineteenth-Century East Africa’, in *Women and Slavery in Africa*, ed. Clare Robertson and Martin Klein (Portsmouth: Heinemann, 1983), 185–219; Marcia Wright, ‘Bwanikwa: Consciousness and Protest among Slave Women in Central Africa, 1886–1911’, in *Women and Slavery in Africa*, ed. Robertson and Klein, 246–67; Hollian Wint, ‘Credible Relations: Indian Finance and East African Society in the Indian Ocean, c. 1860–1940’ (PhD diss. New York University, 2016), 130, 144, 253–78.

36 On the genre and objectives of the Maisha, see Michelle Decker, ‘The “Autobiography” of Tippu Tip: Geography, Genre and the African Indian Ocean’, *Interventions* 17, no. 5 (2015): 744–58.

37 Tippu Tip, *Maisha ya Hamed bin Muhammed el Murjebi yaani Tippu Tip, kwa maneno yake mwenyewe kimefasiriwa na W.H.Whitely* (Nairobi: East African Literature Bureau, 1974, 1st ed: 1959), hereafter: Tippu Tip, Maisha (Whitely), 48–9. ‘Frasila’ is a Swahili unit of weight. One frasila corresponds to roughly 35 pounds or 16 kilos.

38 Tippu Tip, Maisha (Whitely), 26–7.

39 Ibid., 60–1.

40 Ibid., 68–9.

41 Ibid., 76–7.

42 Ibid., 70–3. The Sultanate of Utetera was located in the Maniema region and was a tributary polity subject to the power of the Sultanate of Zanzibar.

43 Ian Georg Deutsch, *Emancipation Without Abolition in German East Africa, c. 1884–1914* (Oxford: Oxford University Press, 2006), 65–6; Jonathon Glassman, ‘The Bondsman’s New Clothes: The Contradictory Consciousness of Slave Resistance on the Swahili Coast’, *Journal of African History* 32, no. 2 (1991): 288–98; on the changing language and meanings of slavery, see Carol Eastman, ‘Service, Slavery and (Swahili) Social Reality’, *Afrikanistische Arbeitspapiere: Schriftenreihe des Kölner Instituts für Afrikanistik* 37 (1994): 88–9.

44 For example, Petro Kilekwa, *Slave Boy to Priest: The Autobiography of Padre Petro Kilekwa* (Westminster: Universities' Mission to Central Africa, 1937); Arthur Cornwallis Madan, ed., and trans., *Kiungani, or, Story and History from Central Africa: Written by Boys in the Schools of the Universities' Mission to Central Africa* (London: Bell & Sons, 1887).

45 Thomas F. McDow, *Buying Time: Debt and Mobility in the Western Indian Ocean* (Athens: Ohio University Press, 2018), chapter 8.

46 Wint, ‘Credible Relations’, chapter 3.

47 David M. Gordon, ‘Precursors to Red Rubber: Violence in the Congo Free State, 1885–1895’, *Past & Present* 236 (2017): 133–68. For a discussion of the treatment of violence as a dominant theme in the regional historiography, see Giacomo Macola, *Una storia violenta. Potere e conflitti nel bacino del Congo, XVIII–XXI secolo* (Rome: Viella, 2021).

48 Murjebi family tree, as discussed by McDow who obtained a copy of Tippu Tip’s genealogy in Arabic from a descendant in 2001, McDow, *Buying Time*, Chapter 5 (Kindle edition, 332ff).

49 Tippu Tip, Maisha (Whitely), 108–9.

50 For example, In Tabora the trader Muhammad bin Juma, of mixed Omani and African descent, married a daughter of *Ntemi* (ruler’s title) Fundikira I of Unyanyembe in the 1840s. For other examples and a discussion of these marriage strategies see David Sperling and Jose Kagabo, ‘The Coastal Hinterland and Interior East Africa’, in *History of Islam in Africa*, ed. Nehemia Levtzion and Randall Pouwels (Athens: Ohio University Press, 2000), 273–302. I am grateful to Salvatory Nyanto for bringing this to my attention.

51 Pius IX was Giovanni Maria Mastai Ferretti. The main evidence on his long papacy is the collection known as Spoglio Pio IX in the Vatican Archives.

52 The term ‘Zouave’ appears to come from groups known as ‘Zwawa’, Algerian Berber-speaking groups who had previously fought for local rulers under the Ottoman Empire. The distinctive elements of the Zouave uniform (baggy trousers, fez beret, etc) were inspired by their Algerian clothing.

53 Christophe Louis Léon Juchault de la Moricière, *Rapport du général de La Moricière à Monseigneur de Mérode, ministre des armées de sa sainteté Pie IX : sur les opérations de l’armée pontificale, contre l’invasion piémontaise dans les Marches de l’Ombrie : accompagné de trois cartes fournies par l’état-major du général Lamoricière* (Paris : Charles Douniol, 1860). On his earlier positions, see : Christophe Louis de la Moricière, *Discours de M. le général de La Moricière député de la Sarthe, dans la discussion du projet de loi relatif aux crédits extraordinaires pour l’Algérie. Session du 9 juin 1847* (Paris : Imprimerie Panckoucke, 1847) ; Christophe Louis de la Moricière, *Projets de colonisation pour les provinces d’Oran et de Constantine … présentés par MM. Les lieutenants généraux de La Moricière et Bedeau* (Paris : Imprimerie Royale, 1848).

54 Athanase Charrette de la Contrie, *Légion des Volontaires de l’Ouest. Commandement supérieur. Appel aux armes*, *19 mars 1871* (Rennes : Imprimerie de Oberthur, 1871).

55 The anti-revolutionary writings of François Athanase Charrette de la Contrie (1763–1796) are extensive, e.g. *Manifeste du charretteCharette*, BNF Gallica, http://catalogue.bnf.fr/ark:/12148/cb37269943v (accessed April 22, 2024).

56 Jeremy Popkin, ‘Religion and Anti-Slavery in Revolutionary France’ (paper presented at the conference ‘Questioning Slavery: Catholic Perspectives’, University of Trier, Germany, June 15–16, 2023).

57 François Renault, *Lavigerie, l’esclavage africain et l’Europe* (Paris : de Boccard, 1971), 369; on the Chouannerie, see Jean-François Paulouin, *La Chouannerie du Maine et Pays adjacents. 1793–1799–1815–1832. Avec la Biographie de plus de 120 Officiers* (Mayenne : Éditions régionales de l’Ouest, 1875); Jacques Duchemin des Cépeaux, *Souvenirs de la Chouannerie* (Laval: H. Godbert, 1855).

58 Letters of Joubert to his mother, Combrée May 8 and May 30, 1860, D.F.-F.2A – L01, as cited in Lourtie, Léopold Joubert, 24 and fn. 33. I consulted the correspondence between Leopold Joubert and members of the Missionaries of Africa at the General Archives of this order in Rome, which hold three letters from Joubert to his mother written after 1880. I could not locate either at the GAMAfr or at the MRAC in Tervuren most of the personal correspondence between Joubert and his relatives cited by Charles Lourtie in his *Le Capitaine Léopold Louis Joubert, 1842–1927* (Neuville-en-Condroz, 2017), the most detailed biography of Joubert. Lourtie explains how he accessed these documents in the early 1870s and states that copies were deposed at the MRAC archives (Lourtie, op.cit, 12–13), but the MRAC does not hold these records. I refer to them as cited in Lourtie’s text.

59 Louis Aimé de Becdelièvre, *Souvenirs de l’armée pontificale* (Paris : Lecoffre fils & Cie, 1867).

60 For an autobiographic account of a volunteer, see Joseph Powell, *Two Years in the Pontifical Zouaves : A Narrative of Travel, Residence, and Experience in the Roman States* (London: R. Washbourne, 1871); see also, Charles A. Coulombe, *The Pope's Legion: The Multinational Fighting Force that Defended the Vatican* (New York: Palgrave McMillan, 2008).

61 Lourtie, *Le Capitaine*, 25.

62 Athanase Charette, *Noces d'argent du régiment des zouaves pontificaux, 1860 – 1885* (Rennes : Oberthur, 1885), 7, citing an article by Léon Aubineau published in *L’Univers*, July 23, 1885.

63 Martin Simpson, ‘From Zouaves Pontificaux to the Volontaires de l'Ouest: Catholic Volunteers and the French Nation, 1860–1910’, *Canadian Journal of History* 53, no. 1 (2018): 1–28.

64 The Zouaves and *Volontaires de l’Ouest* become icons of French Catholic heroism. Joubert’s attitude of self-sacrifice took shape in a culture nourished by the ideals that informed Anatole de Ségur’s *Les Martyrs de Castelfidardo* (Paris: Ambroise Bray, 1861). Similar values are celebrated in memoirs by other Zouaves, including Oscar de Poli’s *Souvenir du bataillon des zouaves pontificaux franco-belges* (Paris: Dupray de la Mahérie, 1861) and in biographies such as Julien Allard’s *Le volontaire Joseph Louis Guerin* (Paris : Douniol, 1862); Charles Du Coëtlosquet, *Theodore Wibaux, Zouave Pontifical et Jesuite* (Lille : Imprimeurs des Facultés Catholiques de Lille, 1885); Robert Oheix, *Joseph Rialan, sergent aux zouaves pontificaux* (Nantes : Forest & Grimaud, 1868). Contemporary religious sermons reinforced these values as their authors, Catholic priests of various ranks, assured their audiences of how the Zouave’s sacrifice pleased God and would receive ample otherworldly compensation: Louis-Edouard Pie, *Eloge funebre des volontaires catholiques, 11 October 1860* (Paris : V. Palmé, 1860); Félix Dupanloup, *Oraison funèbre des volontaires catholiques de l’armée pontificale, morts pour la défense du Saint-Siège, 2 octobre 1860* (Paris : Gatineau, 1860). See Laurent Gruaz, ‘L’extraordinaire chrétien chez les Zouaves pontificaux: Joseph-Louis Guérin (1838–1860) mort en odeur de sainteté’, *Revue d’Histoire des Religions* III (2017): 485–517; Raymond Jonas, *France and the Cult of the Sacred Heart: An Epic Tale for Modern Times* (Berkeley: University of California Press, 2000).

65 Les pères blancs au Sahara, notes, 1872, GAMAfr, diaries, Sahara-Soudan, 1868–1901, 2–3.

66 David Northrup, ‘A Church in Search of a State: Catholic Missions in Eastern Zaïre, 1879–1930’, *Journal of Church and State* 30, no. 2 (1988): 310.

67 For detailed reconstructions of these events by scholars who are also members of the White Fathers order, see Aylward Shorter, *The Cross and Flag in Africa: The White Fathers during the Colonial Scramble, 1892–1914* (Maryknoll, NY: Orbis, 2006); Jean Claude Ceillier and François Richard, *Le cardinal Charles Lavigerie et la campagne antiesclavagiste* (Rome: Société des missionnaires d'Afrique, 2012).

68 Lettre d'Athanase Joubert à un ami, La Chevrottière, April 27, 1879, D. F. – F. 6/3 – n° 3, as cited by Lourtie, *Le Capitaine*, 56, fn. 97.

69 Lettre de Joubert à son frère Athanase, Ancenis, January 14, 1880, D. F. – F. 3A-L.01, as cited by Lourtie, *Le Capitaine*, 57, fn. 98.

70 On the White Fathers’ early relations with Arab elites in Ujiji, see letters from Père Deniaud to Lavigerie, Ujiji, April 12 and November 24, 1879, published in the Missionaries of Africa’s periodic bulletin ‘Les Missions d’Alger’ (hereafter LMA), no. 34 (April 1880): 172–6 and 181–3. The full collection of LMA is held at the GAMAfr.

71 G. Valbert (pseudonym of Victor Cherbuliez), ‘Le Major de Wissman et son second voyage à travers l’Afrique équatoriale’, *Revue des Deux Mondes*, 3e période, no. 105 (1891), 207.

72 Tippu Tip, Maisha (Whitely), 26–9.

73 Ibid., 78–9.

74 Detailed maps of Tippu Tip’s travels are provided in: Stuart Laing, *Tippu Tip: Ivory, Slavery, and Discovery in the Scramble for Africa* (Newport: Medina Publishing ltd, 2017).

75 Verney Lovett Cameron, *Across Afric*a (New York: Harper & Bros, 1877), 281.

76 Tippu Tip, Maisha (Whitely), 112–13.

77 Ibid., 98–9.

78 Ibid., 123–4.

79 ‘The name of Said Barghash has been a powerful support for Europeans in the last year or two […] Tippu Tip, the famous Manyema Arab of whom Stanley speaks, was appointed governor of Tabora by Said Barghash […]’. Letter, J. Faure to Lavigerie, Tabora, May 16, 1883, LMA, GAMAfr, no. 48 (October 1883), 114–8.

80 On a later occasion Tippu Tip went on a joint trade expedition with German ivory trader Mr Giesecke, who was shot. Tippu Tip sent him to be nursed by the White Fathers in Kipalapala and confronted those who shot him: ‘Look here, you have attacked me without any reason. It is not the European you’ve attacked, its me’, Tippu Tip, Maisha (Whitely), 122–3.

81 Tippu Tip, Maisha (Whitely), 92–3.

82 Alexandre LeRoy, *Le Congrès de Bruxelles, L’esclavage Africain*, November 25, 1889, AAV, Segreteria di Stato, 1894, rubr. 204, fasc. 3.

83 Tippu Tip, Maisha (Whitely), 84–7.

84 Ibid., 88–9.

85 When Tippu Tip arrived in Zanzibar after one of his journeys, the Sultan warned him that the British Consul John Kirk was upset about Tippu Tip’s gunpowder trade, and he’d have to pay a fine or spend time in gaol. A compromise was reached, and Tippu Tip spent a couple of days in gaol. The punishment was symbolic, as Tippu Tip was allowed comforts such as a fine room, servants and women during his detention. When he was released, Tippu Tip met the Consul, who allegedly expressed regret for Tippu Tip’s imprisonment, since the latter gave him a letter from Livingstone and made Kirk understand that the Scottish missionary owed his life to him. Tippu Tip, Maisha (Whitely), 34–7.

86 On diplomatic relations between the Al Bu Sa’id sultans and European powers, see Beatrice Nicolini, ‘Muslim Identities of the Indian Ocean: the Ibadi Al Bu Saʾid of Oman during the Eighteenth and Nineteenth Centuries’, in *Muslim Cultures of the Indian Ocean: Diversity and Pluralism, Past and Present*, ed. Stéphane Pradines and Farouk Topan (Edinburgh: Edinburgh University Press, 2023), 67–9; Christine Nichols, *The Swahili Coast. Politics, Diplomacy and Trade on the East African Littoral 1798–1856* (London: Africana Publishing, 1971); Marek Pawełczak, *The State and the Stateless. The Sultanate of Zanzibar and the East African Mainland: Politics, Economy and Society, 1837–1888* (Warszawa: Instytut Historyczny Uniwersytetu Warsawskiego, 2010); Abdul Sheriff, ‘History of Zanzibar to 1890’, *Oxford Research Encyclopedia of African History*, 30–34*.* Published online July 30, 2020 https://doi.org/10.1093/acrefore/9780190277734.013.669 (accessed April 22, 2024).

87 Tippu Tip, Maisha (Whitely), 112–3.

88 Ibid., 124–5.

89 Ibid., 124–5. This refers to the so-called Emin Pasha Relief Expedition, also described in the published diaries of the main European members of the expedition: Major Edmund Musgrave Barttelot, Captain William Grant Stairs, Mr. Arthur Jephson, and Thomas Heazle Parke.

90 Tippu Tip, Maisha (Whitely), 125.

91 Joubert noted: ‘Stanley left with Tippu Tip and 620 Zanzibaris from the Cape to the Congo. His mission appears to be to re-establish the station of Stanley Falls and find Emin Bey … ’, Diary, June 2, 1887, GAMAfr, E.15. On January 25, 1888, when the objectives of Stanley and Tippu Tip’s expedition had become clearer to him, Joubert commented: ‘It would seem that Stanley should establish Tippu Tip – whose people destroyed the station of Stanley Falls in the first place – as chief of this station. The affair is so absurd that I hesitate to believe it’, Diary, January 25, 1888, GAMAfr E.15.

92 Tippu Tip, *Maisha* (Whitely), 128–9 (my italics).

93 Joubert, letter to Lavigerie, St Louis de Mrumbi, October 8, 1890, GAMAfr C10–143.

94 Ibid. (my italics).

95 ‘Those who were redeemed considered themselves and were considered by others as domestic slaves of the missionaries’, *Lavigerie, l’esclavage africain*, vol. I, 203; Northrup, ‘The Ending of Slavery’, 469.

96 Stephen Rockel, ‘“A Nation of Porters”: The Nyamwezi and the Labour Market in Nineteenth Century Tanzania’, *The Journal of African History* 61, no. 2 (2000): 173–95. See also Rockel ‘Caravan Porters of the Nyika’.

97 Tippu Tip, Maisha (Whitely), 18–9. Tippu Tip’s brother with him in this expedition was Muhammad Bin Masoud al-Wardi, Tippu Tip’s maternal half-brother, also known as ‘Kumba Kumba’.

98 Tippu Tip, Maisha (Whitely), 20–1.

99 Cameron, *Across Africa*, 270.

100 Tippu Tip, Maisha (Whitely), 24–5.

101 Ibid., 44–5.

102 Ibid., p. 43–7.

103 Joubert, letter to Lavigerie, report of the trip from Kibanga to Bagamoyo (November 5, 1884 to May 2, 1885), entry of November 22, 1884, GAMAfr C10–132.

104 ‘It starts raining and it’s tiring to walk. Some slave owners are obliged to sell enslaved children who cannot continue walking in the mud’, December 1, 1884.

105 Joubert, Diary, November 22, 1884, GAMAfr, E.15.

106 Joubert, Diary, November 25, 1884, GAMAfr, E.15.

107 Joubert, Diary, November 27, 1884, GAMAfr, E.15.

108 Joubert, Diary, November 27, 1884, GAMAfr, E.15.

109 Charette, *Noces d’argent*, 47, 128.

110 Eriau, *Le Capitaine Joubert*, 13.

111 Fonds Emile Storms, MRAC, HA.01.0017; see also Norman R. Bennett, ‘Captain Storms in Tanganyika: 1882–1885’, *Tanganyika Notes and Records* 54 (1960): 51–63.

112 Emile Banning, *L’Afrique et la conférence géographique de Bruxelles* (Brussels : C. Muquardt, 1876), 123–8. Banning was the Secretary of the Conference where the IAA was founded in September 1876. See also Lavigerie, *Memoir secret*, 4–25.

113 R. Heremans, ‘Lés etablissements de 1'Association Internationale Africaine au lac Tanganika et les Pères Blancs, Mpala et Karema, 1877–1885’, *Annales, Musée Royale de l’Afrique Centrale, Sciences Historiques* 3 (Brussels, 1966).

114 Renault, *Lavigerie, l’esclavage africain*, 272; Heremans, ‘Lés etablissements’, 109.

115 Diary, Mpala, 1885, September; letter of Moinet to fellow brothers, Mpala, September 27, 1885. GAMAfr.

116 François Renault, *Lavigerie, l'esclavage africain*, 363–9. Initially, the Fathers thought that the best candidate for this aspiration would be the Buganda Kingdom under the rule of Kabaka Mutesa (sometimes spelled Mtesa) who was a Christian convert. But religious conflict in Buganda made them divert their attention toward Mpala and Joubert, see also Missionaries of Africa website, https://web.archive.org/web/20111117031911/http://mafrome.org/mpala.htm (accessed April 22, 2024); Jacques Casier, *Le royaume chrétien de Mpala : 1887 – 1893* (Bruxelles: Nuntiuncula, 1987); J. Eriau, *Le capitaine Joubert, chevalier et apôtre, 1842–1927* (Anvers: Fromageau, 1934) ; Northrup, ‘A Church in Search of a State’, 313.

117 Moinet to Cardinal Lavigerie, from Mpala, August 2, 1885, GAMAfr C19–295.

118 Vicariat Apostolique du Tanganyika, La Mission de Karema (1885–1935), 6, GAMAfr, B1 ATM 1.

119 Joubert drew a map of allied and enemy villages in: Diary, April 13, 1887, GAMAFr, E.15. Roberts summarised as follows: ‘The limit of Joubert’s direct authority appears to have been a radius of about three days’ hiking from the mission at the mouth of the Lufuko. Beyond this, many others gave him their allegiance, from the Lake Mweru region in the southwest to beyond the Lukuga River in the north. To these he sent armed support in time of need, and to some even farther afield, powder and an invitation to move closer to him’. Allen F. Roberts, ‘History, Ethnicity and Change in the “Christian Kingdom”’, in *The Creation of Tribalism in Southern Africa*, ed. Leroy Vail (Berkeley: University of California Press, 1989), 198.

120 The White Fathers reported everyday activities in diaries kept in each mission station and usually written by the local superior. On using these diaries as historical sources, see Reuben Loffman, ‘On the Fringes of a Christian Kingdom: The White Fathers, Colonial Rule, and the Báhêmbá in Sola, Northern Katanga, 1909–1960’, *Journal of Religion in Africa* 45, no. 3/4 (2015): 281–2. See also Carol Dickerman, ‘On Using the White Fathers Archives’, *History in Africa* 8 (1981): 320.

121 Joubert, Diary, Mpala, May 5, 1887, GAMAfr E.15.

122 See, for example, Joubert, Diary, Mpala, April 25, 1887, GAMAfr E.15.

123 *Loi sur le meurtre et la vente des esclaves*, Joubert, Diary, Mpala, May 17, 1887, GAMAfr E.15.

124 For example, ‘a couple is condemned to caning for adultery’, Joubert, Diary, June 20, 1887; ‘he who will have hit his chief will face the death penalty’, Joubert, diary, 6 July 1887; ‘punishment for rape: 10 dotis and 100 cane strikes’, Joubert, Diary, Mpala, September 5, 1887, GAMAfr E.15.

125 It would be necessary (…) to do with the slavers what we did with the corsairs of the past, we hung them high and short. This is not the place here, but they should be shot dead: a few executions known, and known afar, would stop them all … very harsh words from my mouth … hard remedy to apply, but it would be effective. Letter of Coulbois to Déguerry, Kibanga, January 2, 1889, Pro-vicariat Haut-Congo, Correspondances des pro-vicaires, François Coulbois, GAMAfr, C19–126.

126 As cited in Renault, *Lavigerie*, *l’esclavage africain*, vol. 1, 386.

127 ‘Reverend Father Coulbois argues that I am in a fake position that compromises the mission’. The letter goes on to explain his views on the necessity to themselves when faced with the hostility of the ‘Arabs’ (and Tippu Tip). Joubert, Letter to Lavigerie, Mpala, February 26, 1888, C10–137; ‘The Reverend Father Coulbois disapproves of this law and fears that it will attract the animosity of the Wangwana … ’ Joubert, Letter to Lavigerie, Mpala, June 1, 1888, C10–138; See also: Joubert, Diary 2, March 25, 1888, GAMAfr, E.15 and Joubert, letter to Lavigerie, Mpala, June 8, 1889, GAMAfr, C 10–140.

128 Captain Joubert's main mission is to protect the missionaries, as well as the personnel and equipment of their stations. He is assisted […] by one or more auxiliaries […] entirely under his orders; is responsible for all matters relating to the military command of the posts of Mpala and Karema and the defence of these posts in the event of an attack; receives ordinary hongos […] of the Chiefs who have placed themselves under his protection; settles disputes that arise among them and ensures their protection […]; is charged with all that concerns the external police of the Stations and has the right to repress mutinies and revolts and to punish the culprits. He will choose […] the most devoted men […] and will train them in the handling of arms … So that it is well established that his action is absolutely different from that of the missionaries, he will have an apartment separate from their house. *Règlement des attributions du capitaine Joubert*, Paris, juillet 1888, GAMAfr, C 10–139.

129 Joubert, Diary, Mpala, May 17, 1887, GAMAfr E.15.

130 Joubert, Diary, Mpala, January 22, 1889, GAMAfr E.15.

131 Joubert, Diary, Mpala, September 19, 1889, GAMAfr E.15.

132 Joubert, Diary, Mpala, September 20, 1889, GAMAfr E.15.

133 Further examples can be found in David Gordon, ‘Slavery and Redemption in the Catholic Missions of the Upper Congo, 1878–1909’, *Slavery & Abolition* 38, no. 3 (2017): 578.

134 Mémoire présenté à Sa Sainteté le Pape Leon XIII sur l’œuvre antiesclavagiste fondée par son éminence le Cardinal Lavigerie, GAMAfr A17.1 (5). See also Jean Perraudin, *Le Cardinal Lavigerie et Leopold II* (Rome: CIPA, 1958). Robert Harms discusses Leopold II’s abolitionist posturing as a ‘ploy to attract British support’, Robert Harms, *Land of Tears: The Exploration and Exploitation of Equatorial Africa* (New York: Basic Books, 2019), 26–7. While relations with Britain were a concern, the Belgian King’s attitudes toward the Congo had many causes. Belgium was a new nation created in 1830. The Prince of Brabant, later King Leopold II, had been keen to demonstrate that his young country could compete with more powerful European empires. He advocated colonial expansion despite arguments against it by anti-colonial liberal economists, Jean Stengers, ‘L’anticolonialisme libérale du XIXe siècle et son influence en Belgique’, *Bulletin des Séances de l’Académie Royale des Sciences d’Outre-mer 1965–2* (Brussels, 1965): 481–521. Leopold II’s imperialist abolitionism developed in the framework of rising Catholic abolitionism and its reverberations in Belgian religious orders, Guy Vanthemsche, *Belgium and the Congo, 1885–1980* (Cambridge: Cambridge University Press, 2012), see particularly 65–99.

135 Lavigerie, *Mémoire présenté à Sa Sainteté le Pape Leon XIII* (my italics).

136 Rumaliza, letter to Coulbois, s.d.; text copied in Diary of Kibanga, March 10, 1889, GAMAfr, original diaries.

137 Joubert, letter to Lavigerie, nd, January 1890, GAMAfr C10–141.

138 Joubert, Diary, October 25, 1892, GAMAfr E.15. See also letter of Joubert Athanase to a friend, Nantes, November 8, 1889, D. F. – F. 8 – L. 17, as cited in Lourtie.

139 Joubert, Diary, September 23, 1891, GAMAfr E.15; see also Joseph Verhoeven, *Jacques de Dixmude l’Africain. Contribution à l’histoire de la société antiesclavagiste belge 1888–1894* (Brussels: Librairie Coloniale, 1929).

140 Joubert, Diary, October 30, 1891, GAMAfr E.15.

141 Joubert, Diary, January 27, 1892, GAMAfr E.15.

142 There is a large literature on all the various aspects of these dynamics, e.g. Norman Bennett, *Arab Versus European: Diplomacy and War in Nineteenth-Century East Central Africa* (New York: Africana, 1986); Philip Gooding, *On the Frontiers of the Indian Ocean World: A History of Lake Tanganyika c. 1830–1890* (Cambridge: Cambridge University Press, 2022), see particularly 170–92 for a discussion of bondage and coercion.

143 European violence was directed against Rumaliza and his allies, who were known. As for Rumaliza’s forces, they had hundreds of firearms and attacked the African allies of the White Fathers: for example, between April and December 1892, they were reported to have killed or captured about ten thousand locals; they posed a threat to Europeans in the region and put their settlements under siege. These events were reported in the publications of various European antislavery societies and discussed in the writings of the Europeans involved. For an overview, see Renault, *Lavigerie, l’esclavage africain*, 428–46. Here I discuss primarily the developments of Belgian colonial violence around Lake Tanganyika, but similar episodes involved other European powers and other groups in the subregion: see Beatrice Nicolini, ‘The 1896 War Against Zanzibar. New Reflections about Imperial Military Power’, *Africana* 30 (2024): 25–57.

144 Alexis-M. G., *Alexis Vrithoff, compagnon des capitaines Jacques et Joubert au Lac Tanganyika (Afrique centrale) : sa jeunesse, son journal de voyage, sa mort glorieuse* (Bruges : Desclée de Brouwer & co., 3rd ed. 1902 (1st ed. 1893)), 133. The author had taught Alexis Vrithoff and was Vice-President of the Société Antiesclavagiste de Namur.

145 Joubert, Diary, April 8, 1892, GAMAfr E.15.

146 Alexandre Delcommune, *Vingt Années de vie africaine : récits de voyages, d’aventures et d’exploration au Congo Belge, 1874–1893*, Vol. 2 (Bruxelles : Vve Ferdinand Larcier, 1922), 3.

147 Alexandre Delcommune, Diary, 16 June 1891, Musée Royal de l'Afrique Centrale (hereafter MRAC), HA.01.0262, 1–6. See lists of Hausa and West African names in the notebooks of Oscar Bodson, MRAC HA.JWB.191.34 (HA.01.0235).

148 Delcommune, Diary, August 3, 1892, MRAC, HA.01.0262, 1–6.

149 Joubert, Diary, August 20, 1892, GAMAfr E.15.

150 Joubert, Diary, August 21, 1892, GAMAfr E.15. The Hausa men had initially refused, but twenty-two of them were successively convinced to join, cf. Saint Louis de Mrumbi, Diary, August 20 and 21, 1892, GAMAfr E15.3. This is not how Delcommune tells the story: see Delcommune, Diary, August 3, 1892, MRAC, HA.01.0262, 4.

151 Arrival of the Delcommune expedition, of the Société du Katanga. (…) At the news of the siege of Albertville, he decides to accompany Captain Joubert to help him save Mr Jacques. The 100 Hausa Muslims of his caravan refuse to march against their co-religionists: they claim that the time of their commitment is over and want to return home: 25 volunteers agree to follow him. Diary of Saint-Louis-du-Mrumbi, August 20, 1892, GAMAfr, original diaries 91/1.

152 The existence of a slave consciousness in Africa at this time is a debated issue that cannot be fully addressed here. My interpretation is that there was no generic ‘slave consciousness’ because, as many historians have noted, ‘slaves’ held many different statuses in this region and were not a unified group with shared interests: see, for example Jonathon Glassman, ‘The Bondsman’s New Clothes’. Slaves could even own slaves, which obviously militated against the formation of a single slave consciousness opposed to a slaveowners’ one, e.g. McMahon, *Slavery and Emancipation in Islamic East Africa: From Honor to Respectability* (Cambridge: Cambridge University Press, 2013), 13. Cooper thinks that ‘a sense of class unity’ developed slowly among the most marginal groups, including the most marginal ex-slaves, Frederick Cooper, *From Slaves to Squatters: Plantation Labor and Agriculture in Zanzibar and Coastal Kenya 1890–1925* (New Haven: Yale University Press, 1980), 172. However, *particular subgroups* among the formerly enslaved *did* acquire a distinctive consciousness and sense of purpose in the fight against slavery. The ‘doctor catechists’ of Tanganyika who worked with the White Fathers were one such group. The so-called Bombay Africans of Freretown and Rabai occupied similar positions: see Samuel Nyanchoga and Michelle Liebst, ‘Rethinking Liberated Africans as Abolitionists: Bombay Africans, Resistance, and Ritual Integration in Coastal Kenya, 1846–1900’, *Esclavages & Post-Esclavages* 10 (2024), forthcoming; Fred Morton, *Children of Ham: Freed Slaves and Fugitive Slaves on The Kenya Coast, 1873 To 1907* (London: Routledge, 1990). The development of anti-slavery activism among persons who had been enslaved was not a prerogative of male slaves nor of Christians: see Elisabeth McMahon, ‘“Siendi” (I Won’t Go): Concubines’ Activism in the Abolition of Slavery in the Zanzibar Protectorate’, *Slavery & Abolition* 41, no. 2 (2020): 395–412.

153 On this group, see Constant Boom, ‘The Doctor-Catechists in Tanganyika: A Study in Evangelism’ (master’s thesis, SOAS, London, 1970).

154 For example, in a letter to the Mother General of the White Sisters, Adrien Atiman talked with pride of the priesthood of his son, Abbot Joseph, and described the latter as ‘Sudanese like his father and Moubandé by mother’. Adrien Atiman, letter to the Mother Superior of the White Sisters, Karema, February 16, 1923, GAMAfr B1 ATM 1.

155 Joubert, Diary, November 16–18, 1896, GAMAfr E.15.

156 Roger Heremans, ‘Joseph Gatchi’, *Bibliographie Belge d’Outre-Mer, Academie Royale des Sciences d’Outre Mer*, Volume 7, 162–3.

157 Adrien Atiman, letter to Father Deguerry, Notre Dame de Karema, October 4, 1889, GAMAfr, B1 ATM 1.

158 Adrien Atiman, letter to Cardinal Lavigerie, Kipalapala, November 10, 1888, GAMAfr, B1 ATM 1.

159 Adrien Atiman, letter to Father Deguerry, Notre Dame de Karema, February 17, 1891, GAMAfr, B1 ATM 1.

160 Adrien Atiman, letter to the Mother Superior of the White Sisters, Karema, July 25, 1923, GAMAfr, B1 ATM 1; underlining in the original.

161 Sayyida Salme/Emily Ruete has been the subject of debate as a square not far from her former home in Hamburg was named after her in 2020, a gesture aimed at celebrating ‘as a model immigrant of her time’ an intellectual woman of colour from Zanzibar, but the decision was reversed because she was found to have benefited from slavery and to have entertained racist views, see Klaus Neuman, ‘Cancelling Bismarck: Black Lives Matter, a princess from Zanzibar and Germany’s “Memorial Hygiene”’, *Inside Story*, published online November 18, 2020, https://insidestory.org.au/cancelling-bismarck-neumann/ (accessed April 22, 2024).

162 Farouk Topan, ‘Transcending Boundaries: Sayyida Salme/Emily Ruete and Siti binti Saad’, in *Muslim Cultures of the Indian Ocean: Diversity and Pluralism, Past and Present*, ed. Stephane Pradines and Farouk Topan (Edinburgh: Edinburgh University Press, 2023), 91–115.

163 Sayyida Salme/Emily Ruete, *An Arabian Princess between Two Worlds. Edited with an introduction of E.van Donzel*, trans. Ulrich Haarmann (Leiden: Brill, 1993), 330 (my italics).

164 Ibid., 330–1, footnote 142.

165 Michelle Liebst, *Labour and Christianity in the Mission: African Workers in Tanganyika and Zanzibar, 1864–1926* (Woodbridge: James Currey, 2021), 8–9, 75–100.

166 David Northrup, ‘Slavery and Forced Labour in the Eastern Congo 1850–1910’, in *Slavery in the Great Lakes Region*, ed. Médard and Doyle, 115–6; coercive labour arrangements went on into colonial and postcolonial periods, see Reuben Loffman, ‘Belgian Rule and its Afterlives: Colonialism, Developmentalism, and Mobutism in the Tanganyika District, Southeastern DR-Congo, 1885–1985’, *International Labor and Working Class History* 92 (2017): 47–68.

167 For examples, see Salvatory Nyanto and Felicitas Becker, ‘In Pursuit of Freedom: Oaths, Slave Agency, and the Abolition of Slavery in Western Tanzania 1905–1930’, *Law and History Review* 42, no. 1: 119–41; Nyanchoga and Liebst, ‘Rethinking Liberated Africans as Abolitionists’; Margot Luyckfasseel, ‘Female Options: Slavery, Marriage, and Women’s Social Mobility among the Wangwana in Central-East Congo at the Turn of the Twentieth Century’ (paper presented at online seminar of the Project ‘Aftermaths of Slavery in East Africa’ (ASEA), June 22, 2023); Elisabeth McMahon, ‘“Siendi (I won’t go)”’.

168 This article re-joins Richard Reid’s suggestion to focus more on African agency in the ‘scramble’, Richard Reid, ‘Africa’s Revolutionary Nineteenth Century and the Idea of the “Scramble”’, *The American Historical Review* 126, no. 4 (2021): 1424–47.

169 Sanjay Subrahmanyam, ‘Connected Histories: Notes Toward a Reconfiguration of Early Modern Eurasia’, *Modern Asian Studies* 31, no. 3 (1997): 735–62; Tonio Andrade, ‘A Chinese Farmer, Two African Boys and a Warlord: Toward a Global Microhistory’, *Journal of World History* 21, no. 4 (2010): 573–91; John-Paul Ghobrial, ‘Introduction: Seeing the World as a Micro-historian’, *Past and Present*, Supplement 14 (2019): 1–22.

170 Romain Bertrand, ‘Where the Devil Stands: A Micro-historical Reading of Empires as Multiple Moral Worlds (Manila-Mexico, 1577–1580)’, *Past and Present*, Supplement 14 (2019): 83–109.

171 Edoardo Grendi, ‘Micro-analisi e storia sociale’, *Quaderni Storici* 12, no. 35/2 (1977): 512.

172 George Marcus, ‘Ethnography in/of the World system: the Emergence of Multisited Ethnography’, *Annual Review of Anthropology* 24 (1995): 109.

173 Carlo Ginzburg and Carlo Poni, ‘Il nome e il come: scambio ineguale e mercato storiografico’, *Quaderni storici* 14, no. 40/1 (1979): 181–90.

